# (*R*/*S*)-lactate/2-hydroxybutyrate dehydrogenases in and biosynthesis of block copolyesters by *Ralstonia eutropha*

**DOI:** 10.1007/s00253-023-12797-6

**Published:** 2023-09-29

**Authors:** Shizuru Ishihara, Izumi Orita, Ken’ichiro Matsumoto, Toshiaki Fukui

**Affiliations:** 1 School of Life Science and Technology, Tokyo Institute of Technology, B-37 4259 Nagatsuta, Midori-ku, Yokohama, 226-8501 Japan; 2 Division of Biotechnology and Macromolecular Chemistry, Graduate School of Engineering, Hokkaido University, N13W8, Kita-ku, Sapporo, 060-8628 Japan

**Keywords:** *Ralstonia eutropha*, Polyhydroxyalkanoates, Block copolymer, 2-Hydroxybutytate, Lactate dehydrogenase, Metabolic engineering

## Abstract

**Abstract:**

Bacterial polyhydroxyalkanoates (PHAs) are promising bio-based biodegradable polyesters. It was recently reported that novel PHA block copolymers composed of (*R*)-3-hydroxybutyrate (3HB) and (*R*)-2-hydroxybutyrate (2HB) were synthesized by *Escherichia coli* expressing PhaC_*AR*_, a chimeric enzyme of PHA synthases derived from *Aeromonas caviae* and *Ralstonia eutropha*. In this study, the sequence-regulating PhaC_*AR*_ was applied in the natural PHA-producing bacterium, *R. eutropha*. During the investigation, (*R*/*S*)-2HB was found to exhibit strong growth inhibitory effects on the cells of *R. eutropha*. This was probably due to formation of excess 2-ketobutyrate (2KB) from (*R*/*S*)-2HB and the consequent l-valine depletion caused by dominant l-isoleucine synthesis attributed to the excess 2KB. Deletion analyses for genes of lactate dehydrogenase homologs identified cytochrome-dependent d-lactate dehydrogenase (Dld) and [Fe-S] protein-dependent l-lactate dehydrogenase as the enzymes responsible for sensitivity to (*R*)-2HB and (*S*)-2HB, respectively. The engineered *R. eutropha* strain (*phaC*_*AR*_^+^, *ldhA*_*Cd*_*-hadA*_*Cd*_^+^ encoding clostridial (*R*)-2-hydroxyisocaproate dehydrogenase and (*R*)-2-hydoroxyisocaproate CoA transferase, ∆*dld*) synthesized PHA containing 10 mol% of 2HB when cultivated on glucose with addition of sodium (*RS*)-2HB, and the 2HB composition in PHA increased up to 35 mol% by overexpression *phaC*_*AR*_. The solvent fractionation and NMR analyses showed that the resulting PHAs were most likely to be block polymers consisting of P(3HB-*co*-3HV) and P(2HB) segments, suggesting that PhaC_*AR*_ functions as the sequence-regulating PHA synthase independently from genetic and metabolic backgrounds of the host cell.

**Key points:**

- *(R/S)-2-hydroxubutyrates (2HB) caused l-valine deletion in Ralstonia eutropha*
- *(R)- and (S)-lactate/2HB dehydrogenases functional in R. eutropha were identified*
- *The engineered R. eutropha synthesized block copolymers of 2HB-containing polyhydroxyalkanoates on glucose and 2HB*

**Graphical Abstract:**

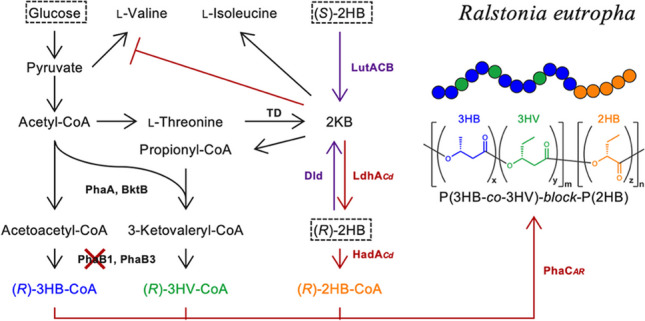

**Supplementary Information:**

The online version contains supplementary material available at 10.1007/s00253-023-12797-6.

## Introduction

Environmental pollution caused by plastic wastes is now becoming more serious on a global scale (Narancic et al. 2018; Waring et al. 2018). Polyhydroxyalkanoates (PHAs), which are biopolyesters accumulated within microbial cells as a carbon and energy storage, are eco-friendly alternatives to petroleum-based plastics because they can be produced from biomass feedstocks and show high biodegradability not only in soil but also fresh and sea water environments (Guzik et al. 2020; Miyahara et al. 2020). Poly((*R*)-3-hydroxybutyrate) [P(3HB)] is the most abundant PHA in nature. In general, P(3HB) is synthesized from acetyl-CoA through three consecutive reactions catalyzed by β-ketothiolase (PhaA), NADPH-dependent acetoacetyl-CoA reductase (PhaB), and PHA synthase (PhaC). It is also known that PHA-producing microbes potentially synthesize copolymers composed of two or more hydroxyalkanoate units. Biosynthesis of PHA copolymers by addition of precursor compounds into the media and metabolic and enzyme engineering approaches have been extensively studied in order to overcome the stiff and hard properties of P(3HB) homopolymer.

PHA synthase, the key enzyme in the PHA biosynthesis, catalyzes polymerization of (*R*)-3-hydroxyacyl (3HA)-CoAs *via* terminal extension of growing polymer chain (Neoh et al. 2022). PHA synthases belonged to α/β-hydrolase superfamily and classified into four classes based on the subunit composition and substrate specificity. The class I and II synthases are both single-subunit enzymes but show distinctly different substrate specificity from each other. The class I enzymes, including that from *Ralstonia eutropha* (*Cupriavidus necator*) H16 (PhaC_*Re*_), are specific to short-chain-length (*R*)-3HA-CoAs from C_3_ to C_5_, while the class II synthases derived from *Pseudomonas* spp. exhibit polymerization activity towards medium-chain-length (*R*)-3HA-CoAs of C_6_ and the longer. Some PHA synthases exceptionally mediate polymerization of both short- and medium-chain-length (*R*)-3HA-CoAs, such as the class I PHA synthase from *Aeromonas caviae* FA440 (PhaC_*Ac*_) polymerizing (*R*)-3HA-CoAs of C_4_-C_6_ (Fukui and Doi 1997), and the class II enzyme from *Pseudomonas* sp. 61-3 having broad substrate specificity to 3HA-CoAs from C_4_ to C_12_ (Matsusaki et al. 1998). PHA synthases often accept not only 3HA-CoAs but also 4-hydroxyacyl-CoAs and 5-hydroxyacyl-CoAs (Taguchi and Matsumoto 2021), whereas no natural synthase capable of accepting 2-hydroxyalkanoate (2HA) units has been known so far. In 2008, the S325T/Q481K double mutant of PHA synthase derived from *Pseudomonas* sp. 61-3 (named as PhaC_STQK_) was found to exhibit copolymerization activity to (*R*)-2HA-CoAs with (*R*)-3HB-CoA (Taguchi et al. 2008), expanding the structural range of microbial polyesters. Recombinant strains of *Escherichia coli* harboring phaC_STQK_ synthesized PHA copolymers of d-lactate and (*R*)-3HB where the highest d-lactate fraction reached up to 97 mol% (Nduko et al. 2013; Shozui et al. 2010; Shozui et al. 2011). It was further demonstrated that (*R*)-2-hydroxybutyryl (2HB)-CoA and glycolyl-CoA were also accepted as substrates for PhaC_STQK_ (Taguchi and Matsumoto 2021).

Unlike DNA and protein synthesis, PHA synthase-mediated polymerization occurs with template-independent manner that results in random distribution of the comonomer units in the polyester chain. This random polymerization property of PHA synthase is one of the limitations in the biological polyester synthesis, because synthesis of sequence-regulated copolymers, often showing altered properties from the corresponding random copolymers (Kumar et al. 2001), had been difficult by using PHA synthases. Recently, a chimeric PHA synthase consisting of *N*- and *C*-terminal regions of PhaC_*Ac*_ and PhaC_*Re*_, respectively, was constructed aiming to create an engineered synthase compatibly possessing the broad substrate specificity of PhaC_*Ac*_ and high polymerization activity of PhaC_*Re*_ (Matsumoto et al. 2009). In vivo characterization of the chimeric PhaC (designated PhaC_*AR*_) in *E. coli* exhibited the polymerization activity to not only (*R*)-3HA-CoAs but also (*R*)-2HA-CoAs. It should be further noted that the resulting PHAs synthesized by PhaC_*AR*_ were block copolymers comprising of (*R*)-3HA-rich and (*R*)-2HA-rich segments, unlike the copolymers synthesized by PhaC_STQK_ (Matsumoto et al. 2009; Matsumoto et al. 2018; Arai et al. 2020; Satoh et al. 2022). One of the block copolymers, poly((*R*)-2HB-*block*-(*R*)-3HB) [P(2HB-*b*-3HB)], was demonstrated to show elastomer-like properties that were not seen for the random copolymer, poly((*R*)-2HB-*random*-(*R*)-3HB) [P(2HB-*ran*-3HB)] (Kageyama et al. 2021). PhaC_*AR*_ is thus considered as a sequence-regulating PHA synthase.

*R. eutropha* H16 is a well-studied PHA-producing bacterium. There are vigorous studies for biochemical and molecular biological analyses regarding PHA biosynthesis by *R. eutropha*, as well as metabolic engineering of this bacterium focusing on efficient production of useful PHA copolymers such as flexible poly((*R*)-3HB-*co*-(*R*)-3-hydroxyhexanoate) [P(3HB-*co*-3HHx)] (Mifune et al. 2010; Insomphun et al. 2014; Insomphun et al. 2015; Zhang et al. 2019). The genome analysis of *R. eutropha* clarified that the 7.4 Mbp genome contains many genes related to metabolisms of fatty acids and carboxylic acids, such as 31 isologs of acyl-CoA synthetase/CoA ligase, 65 isologs of acyl-CoA dehydrogenase, 22 isologs of β-ketothiolase, and so on (Pohlmann et al. 2006), suggesting more complexed and robust metabolisms of *R. eutropha* towards acyl moieties when compared to *E. coli*.

This study investigated the polymerization property of PhaC_*AR*_ in *R. eutropha* having quite different genetic and metabolic backgrounds from *E. coli*, since the sequence-regulating polymerization by PhaC_*AR*_ has been shown only in *E. coli* as the host bacterium so far. During the research, we observed high growth toxicity of 2HB to the cells of *R. eutropha*, and obtained new knowledge with respect to 2HB metabolism in this bacterium. Structural analyses of the 2HB-containing PHAs synthesized by the PhaC_*AR*_-equipped strains with 2HB supplementation strongly suggested the occurrence of the block copolymerization phenomenon in *R. eutropha*.

## Materials and methods

### Bacterial strains and plasmids

The strains and plasmids used in this study are listed in Table 1. *E. coli* strains DH5α and S17-1 were used as hosts for general genetic engineering and transconjugation, respectively. *R. eutropha* strains were routinely cultivated at 30 °C in a nutrient-rich (NR) medium containing 1% (w/v) bonito extract (Kyokuto, Tokyo, Japan), 1% (w/v) polypeptone, and 0.2% (w/v) yeast extract dissolved in tap water. *E. coli* strains were cultivated at 37 °C in a Lysogeny broth (LB) medium. Kanamycin (100 μg/mL for *E. coli* and 250 μg/mL for *R. eutropha* strains) or ampicillin (100 μg/mL for *E. coli*) was added into the medium when necessary.

**Table 1:** Strains and plasmids used in this study

Strain or plasmid	Relevant maker	Source
*Escherichia coli*		
DH5α	F^−^, Φ80d*lacZ∆*M15, ∆(*lacZYA*-*argF*)U169, *deoR*, *recA1*, *endA1*, *hsdR17*(r_k_^−^,m_k_^+^), *phoA*, *supE44*, l^−^, *thi*-1, *gyr*A96, *relA1*	Takara Bio
S17-1	*thi*, *pro*, *hsdR*, *recA*, chromosomal RP4, Tra^+^, Tmp^r^, Str/Spc^r^	Simon et al. 1983
*Ralstonia eutropha*		
H16	Wild type	DSM 428
NSDG-GGDB1	H16 derivative; ∆*phaC*::*phaC*_NSDG_, ∆*nagR*, *nagE*_GR_, *P*_*A2858*_-*glpFK*_*Ec*_-*h16*_*A2858*, ∆*phaB1*	Zhang et al. 2019
IF001	NSDG-GG∆B1 derivative; ∆*phaC*_NSDG_::*phaC*_*AR*_	This study
IF002	IF001 derivative; *phaP1*::*ldhA*_*Cd*_-*hadA*_*Cd*_	This study
IF005	IF001 derivative; ∆*h16*_*A0666*	This study
IF012	IF001 derivative; ∆*h16*_*B0460*	This study
IF013	IF001 derivative; ∆*h16*_*B1817*	This study
IF014	IF001 derivative; ∆*h16*_*A3091*	This study
IF015	IF001 derivative; ∆*h16*_*B0093*-*B0092*-*B0091*	This study
IF016	IF001 derivative; ∆*h16*_*A3091*, ∆*h16*_*B0093*-*B0092*-*B0091*	This study
IF017	IF002 derivative; ∆*h16*_*A3091*	This study
IF018	IF002 derivative; ∆*h16*_*B0093*-*B0092*-*B0091*	This study
IF019	IF002 derivative; ∆*h16*_*A3091*, ∆*h16*_*B0093*-*B0092*-*B0091*	This study
IF023	IF001 derivative; ∆*h16*_*A1681*-*A1682*	This study
Plasmids		
pTTQ19	pMB1 ori, Amp^r^, *lacZ*α, *lacI*^*q*^, *P*_*tac*_	Stark 1987
pTTQ-ldhAhadA_*Cd*_	pTTQ19 derivative; *ldhA*_*Cd*_*-hadA*_*Cd*_ (codon-optimized for *E. coli*)	Mizuno et al. 2018
pBluescript II KS (+)	ColE1 ori, Amp^r^, *lacZ*α, *P*_*lac*_	Agilent Technologies
pBSP_*Re*_phaC_*AR*_pct	pBluescript II KS (+) derivative; *P*_*phaC*_, *phaC*_*AR*_, *pct*_*Me*_	Matsumoto et al. 2018
pK18mobsacB	pMB1 ori, RP4mob, Km^r^, modified *sacB*, *lacZ*α	Schäfer et al. 1994
pK18ms-C61-3EDQKAB’	pK18mobsacB derivative; ∆*phaC*::*phaC*_EDQK_	Laboratory stock
pK18ms-C_*AR*_AB’	pK18ms-C61-3EDQKAB’ derivative; ∆*phaC*::*phaC*_*AR*_	This study
pK18ms-P1udP1J	pK18mobsacB derivative; *phaP1*::*phaJ*_*Ac*_	Kawashima et al. 2015
pK18ms-P1udP1-ldhA_*Cd*_hadA_*Cd*_	pK18ms-P1udP1J derivative; *phaP1*::*ldhA*_*Cd*_-*hadA*_*Cd*_	This study
pK18ms-A0666ud1000	pK18ms derivative; *h16*_*A0666 del*	This study
pK18ms-B0460ud980	pK18ms derivative; *h16*_B*0460 del*	This study
pK18ms-B1817ud980	pK18ms derivative; *h16*_*B1817 del*	This study
pK18ms-A3091ud980	pK18ms derivative; *h16_A3091 del*	This study
pK18ms-B0093-B0092-B0091ud980	pK18ms derivative; *h16_B0093*-*B0092*-*B0091 del*	This study
pK18ms-A1681-A1682ud900	pK18ms derivative; *h16_A1681*-*A1682 del*	This study
pBBR1MCS-2	Broad host range plasmid; Km^r^, *mob*, *P*_*lac*_, *lacZ*α	Kovach et al. 1995
pBPP	pBBR1MCS-2 derivative; *P*_*phaP1*_, *T*_*rrnB*_	Fukui et al. 2011
pBPP-*phaC*_*AR*_	pBPP derivative; *phaC*_*AR*_	This study

*Ec*, *Escherichia coli*; *Ac*, *Aeromonas caviae*; *Cd*, *Clostridioides difficile*; *Me*, *Megasphaera elsdenii*

*del*: Homologous regions for deletion of the target gene by homologous recombination

### Plasmid and strain constructions

DNA manipulations were carried out according to standard procedures. PCR reactions were performed with KOD DNA polymerase variants purchased from Toyobo (Osaka, Japan). The sequences of oligonucleotide primers used in this study are shown in supplementary Table S1.

Modifications of the chromosomes of *R. eutropha* were performed by homologous recombination using pK18mobsacB (Schäfer et al. 1994)-based suicide vectors. Generally, a tandem of up- and down-stream homologous regions (~1000 bp-length) flanking to the target locus was cloned into pK18mobsacB, and the resulting plasmid was used for gene deletion. For gene insertion, a DNA fragment of the gene in interest was inserted between the up- and down-stream regions in the vector, followed by homologous recombination by using the construct. The coding region of *phaC*_*AR*_ was amplified by using a primer set of phaCar_Fw/phaCar_Rv with pBSP_*Re*_phaC_*AR*_pct (Matsumoto et al. 2018) as a template. A fragment containing *ldh*_*Cd*_-*hadA*_*Cd*_ was amplified by using hadA-inf_Fw/hadA-inf_Rv and pTTQ-ldhAhadA_*Cd*__opt (Mizuno et al. 2018) as a primer set and template, respectively. A broad-host-range vector pBPP, which has been constructed for gene expression in *R. eutropha* under the control of *phaP1* promoter (Fukui et al. 2011), was used for overexpression of *phaC*_*AR*_.

Transformation of *R. eutropha* was carried out by transconjugation using *E. coli* S17-1 harboring the mobilizable plasmid as a donor. In the case of pop-in-pop-out recombination using a pK18mobsacB-based suicide vector, the transformants with the desired genotype were identified by appropriate PCR and isolated, as described previously (Mifune et al. 2010).

### PHA production

*R. eutropha* strains were cultivated in a 100 mL phosphate-limited mineral salt medium composed of 0.2 g of NH_4_Cl, 0.087 g of K_2_HPO_4_, 0.02 g of MgSO_4_·7H_2_O, and 0.1 mL of trace-element solution (Kato et al. 1996) in 100 mL of 40 mM 3-(*N*-morpholino)propansulfonate (MOPS) buffer (pH 7.2). A filter-sterilized solution of glucose was added to the medium with the final concentration of 2% (w/v) as a carbon source. Other supplements were added into the medium also from the filter-sterilized stock solution with the final concentrations as indicated in the text. Kanamycin (final concentration; 100 μg/mL) was added when necessary. The cells grown at 30 °C for 72 to 120 h with reciprocal shaking (120 strokes/min) were harvested, washed once with cold deionized water, and then lyophilized. The cellular PHA content and composition were determined by gas chromatography (GC) after direct methanolysis of the dried cells in the presence of 15% sulfuric acid as described previously (Kato et al. 1996). Intracellular PHA was extracted from the lyophilized cells by chloroform with stirring at room temperature for 72 h, and then purified by reprecipitation using cold methanol.

### ^1^H and ^13^C NMR analyses and solvent fractionation

The extracted PHA was dissolved in chloroform-*d* containing 0.05% tetramethylsilane and was subjected to ^1^H and ^13^C NMR analyses with 400-MR NMR spectrometer (Agilent, California, USA). Solvent fractionation of the PHA copolymer synthesized by recombinant *R. eutropha* was performed as below. A total of 30–50 mg of the purified PHA was dissolved in 20 mL chloroform with stirring for 72 h at room temperature, then the solution was mixed with 200 mL tetrahydrofuran (THF). The mixture was stirred for 18 h at 4 °C. The THF-soluble fraction was obtained by passing through a polytetrafluoroethylene membrane filter, and the polymer was recovered by reprecipitation with methanol. The residue on the membrane was dried up and collected as the THF-insoluble polymer fraction (Matsumoto et al. 2018).

## Results

### Introduction of PhaC_*AR*_ and a pathway for 2HB-CoA formation into *R. eutropha*

**Fig. 1:**
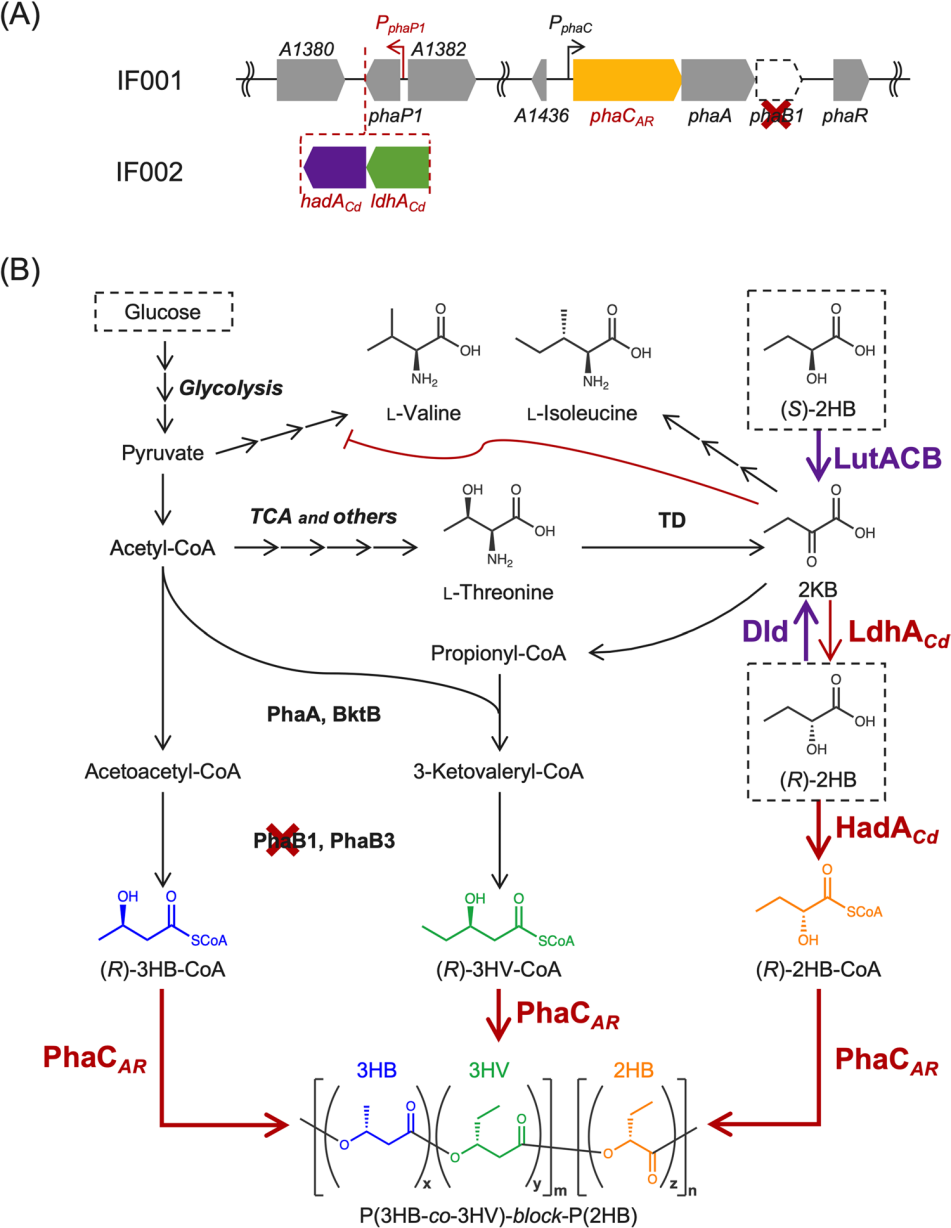
**A** The gene organization of *pha* loci on the chromosome 1 of *R. eutropha* strains IF001 and IF002, **B** metabolic pathway for biosynthesis of 2HB-containing PHA in *R.eutropha*. PhaP1, PHA granule-associated protein (phasin); PhaR, transcriptional regulator for phasin expression; PhaC_*AR*_, chimeric PHA synthase consisting of *N*- and *C-*terminal regions of PHA synthase from *A. caviae* and *R. eutropha*, respectively; PhaA and BktB, β-ketothiolases; PhaB1 and PhaB3, NADPH-dependent acetoacetyl-CoA reductases; LdhA_*Cd*_ and HadA_*Cd*_, (*R*)-2-hydoroxyisocaproate dehydrogenase and (*R*)-2-hydoroxyisocaproate CoA transferase, respectively, derived from *C. difficile* (codon-optimized for *E. coli*); A1380, pyruvate formate-lyase-activating enzyme; A1382, histone deacetylase-family protein; A1436, conserved hypothetical protein; TD, l-threonine dehydratase; Dld, cytochrome-dependent d-lactate dehydrogenase; LutACB, [Fe-S] cluster protein-dependent l-lactate utilization system

*phaC*_*AR*_, the gene of the chimeric PHA synthase, was inserted into chromosome 1 of *R. eutropha* strain NSDG-GG∆B1 to obtain the strain IF001 (Fig. 1A). This was achieved by replacing *phaC*_NSDG_ (encoding N149S/D171G mutant of PHA synthase derived from *A. caviae*) in NSDG-GG∆B1 with *phaC*_*AR*_ by homologous recombination, where the parent strain has been engineered to assimilate glucose (Orita et al. 2012) and glycerol (Fukui et al. 2014) as well as weaken (*R*)-3HB-CoA formation by deletion of *phaB1*. It was expected that the deletion of *phaB1* potentially led to relative increase in comonomer fraction other than (*R*)-3HB in PHA (Zhang et al. 2019).

Further modification was aimed to supply (*R*)-2HB-CoA from 2-ketobutyrate (2KB) generated by deamination of l-threonine in l-isoleucine biosynthesis pathway (Fig. 1B). It has been reported that (*R*)-2-hydroxyisocaproate dehydrogenase (LdhA) and (*R*)-2-hydoroxyisocaproate CoA transferase (HadA) derived from *Clostridium difficile* (currently *Clostridioides difficile*) (Kim et al. 2006) were functional in the formation of (*R*)-2HB-CoA from 2KB for biosynthesis of 2HB-containing PHAs by engineered *E. coli* (Sudo et al. 2020; Mierzati et al. 2020). Here, a tandem of *ldhA*_*Cd*_-*hadA*_*Cd*_, codon-optimized for *E. coli*, was inserted at downstream of *phaP1* encoding a PHA granule-associated protein (phasin) on chromosome 1 of *R. eutropha*, as shown in Fig. 1A (the strain IF002). As the expression of *phaP1* by *phaP* promoter (*P*_*phaP*_) was derepressed by mobilization of PhaR regulator from DNA onto surface of PHA granules (Pötter et al. 2002), the heterologous *ldhA*_*Cd*_-*hadA*_*Cd*_ genes were expected to be highly expressed during PHA accumulation phase.

### Inhibitory effect of 2HB on growth of *R. eutropha* and restoration by l-valine

**Fig. 2:**
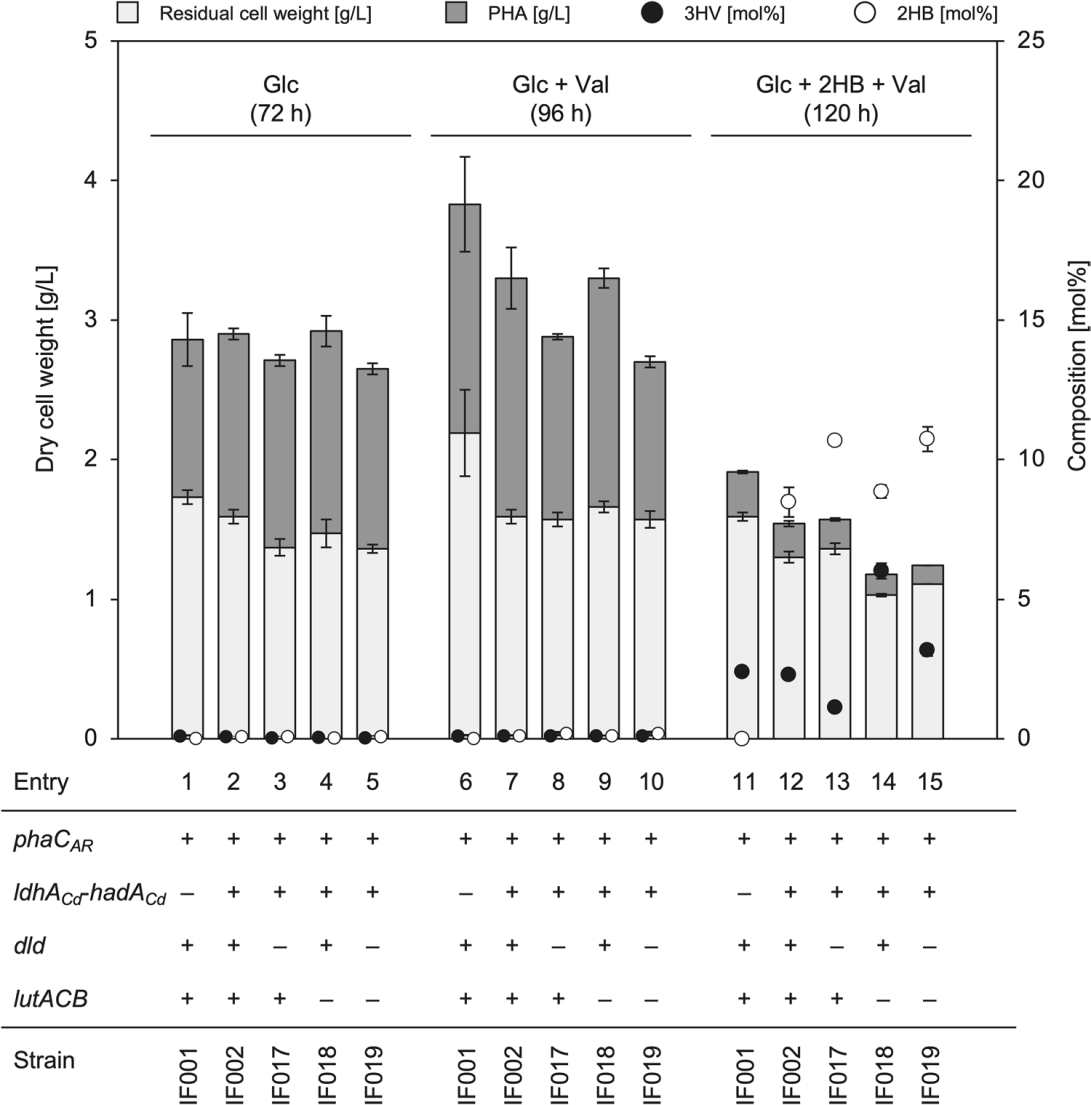
PHA biosynthesis by engineered strains of *R. eutropha* on 2% (w/v) glucose (entries 1 to 5), 2% (w/v) glucose with 0.05% (w/v) l-valine (entries 6 to 10), and 2% (w/v) glucose with 0.25% (w/v) sodium (*RS*)-2HB and 0.05% (w/v) l-valine (entries 11 to 15). The residual cell weight and PHA production are shown in gray and dark gray bars, respectively, and 3HV and 2HB compositions are shown as black and white circles, respectively. The cells were cultivated in a 100 mL phosphate-limited mineral salt medium containing the additives described above for 72–120 h at 30 °C (triplicate)

PHA biosynthesis properties of the resulting strains IF001 and IF002 were examined in a mineral salt medium containing 2% (w/v) glucose as a sole carbon source. Taking into consideration that nitrogen limitation usually represses amino acid biosynthesis and 2KB is an intermediate in l-isoleucine biosynthesis, phosphate limitation using MOPS medium was applied for induction of PHA synthesis. These strains accumulated PHA containing small fractions of 3-hydroxyvalerate (3HV) unit (~0.1 mol%) with cellular contents of 39–45 wt% of the dry cell weight (entries 1 and 2 in Fig. 2 and supplementary Table S2). Unexpectedly, only trace amount of 2HB unit (< 0.1 mol%) was detected in PHA synthesized by IF002.

**Fig. 3:**
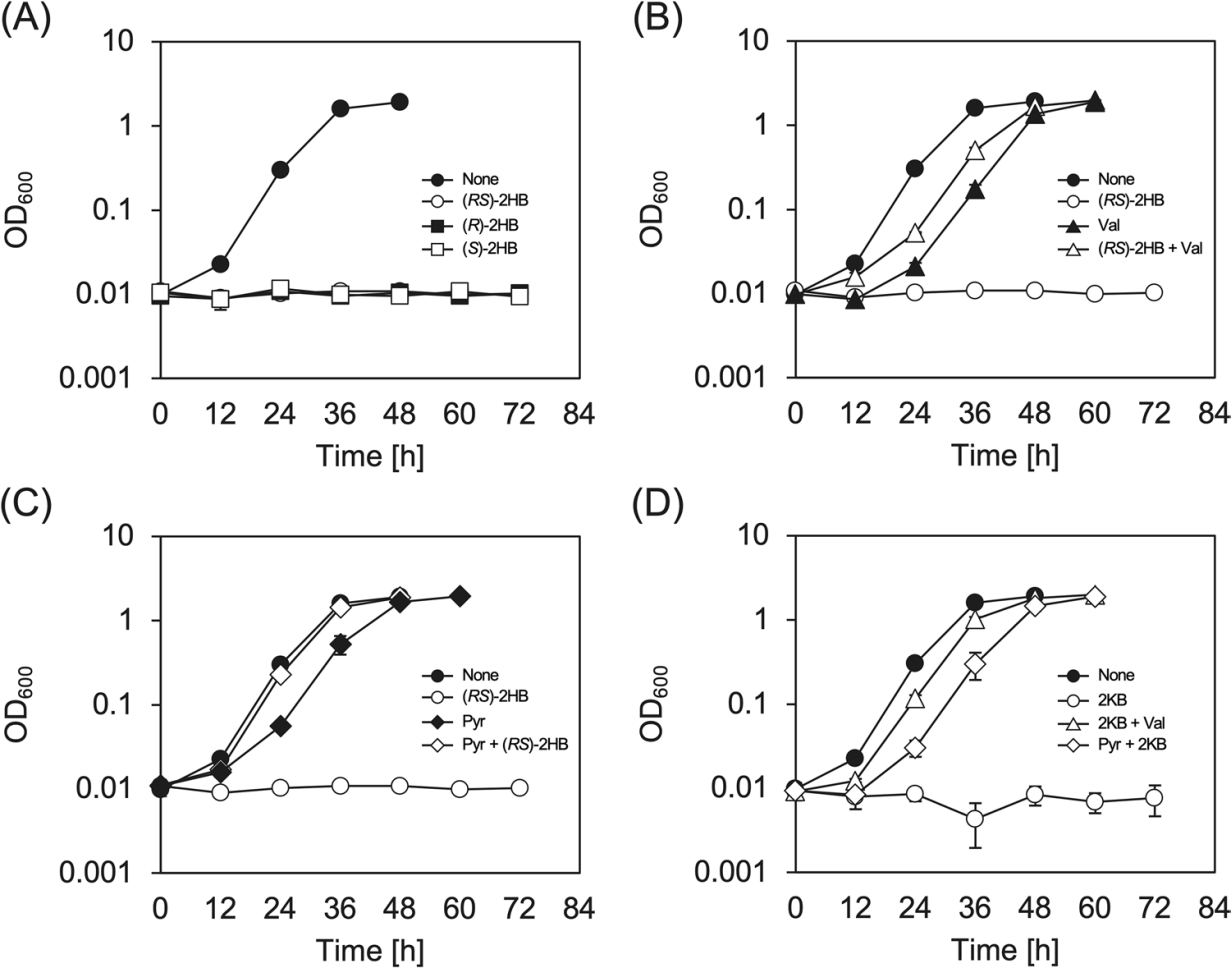
Growth inhibition of *R. eutropha* IF001 by (*R*/*S*)-2HB (**A**) and the restoration by l-valine (**B**) or pyruvate (**C**), and growth inhibition of *R. eutropha* by 2KB and the restoration by l-valine or pyruvate (**D**). The cells were cultivated in a 3 mL phosphate-limited mineral salt medium containing 2% (w/v) glucose for 72 h at 30 °C with reciprocal shaking (170 strokes/min) (triplicate). Supplementations shown in each panel are 0.005% (w/v) sodium (*R*)-2HB, 0.005% (w/v) sodium (*S*)-2HB, 0.01% (w/v) sodium (*RS*)-2HB, 0.01% (w/v) sodium 2KB, 0.05% (w/v) l-valine, and 0.8% (w/v) sodium pyruvate

We then examined cultivation of the strains in the glucose medium supplemented with 0.25% (w/v) sodium (*RS*)-2HB as the precursor in order to confirm polymerization activity of PhaC_*AR*_ towards 2HB-CoA in *R. eutropha*. However, both the strains showed no growth in the presence of (*RS*)-2HB. When the strain IF001 was cultivated in the media containing lower concentrations of (*R*)-2HB, (*S*)-2HB, or (*RS*)-2HB (0.005–0.01% (w/v)), the cells also could not grow under all the conditions examined (Fig. 3A). These results demonstrated a strong inhibitory effect of (*R*/*S*)-2HB on the cell growth of *R. eutropha* regardless stereo-configuration of the 2-hydroxyl group. It was assumed that 2KB, formed by dehydrogenation of (*R*/*S*)-2HB, gave some effects on metabolisms of branched-chain amino acids and consequent toxicity, because 2KB is an intermediate in biosynthesis of l-isoleucine from l-threonine (supplementary Fig. S1). We found that, among branched-chain amino acids (l-isoleucine, l-valine, and l-leucine), only l-valine could restore the cell growth in the presence of 0.01% (*RS*)-2HB (Fig. 3B). Pyruvate, a precursor of l-valine, also restored the growth in the presence of (*RS*)-2HB when added with high concentration of 0.8% (w/v) (Fig. 3C). Additionally, supplementation of 0.01% 2KB also significantly impaired the growth of IF001 with similar degree to (*R*/*S*)-2HB, and the growth restoration by l-valine or pyruvate was again observed (Fig. 3D). These results strongly supported that the growth inhibition by (*R*/*S*)-2HB was due to depletion of l-valine caused by inhibitory effects of 2KB generated from (*R*/*S*)-2HB on l-valine biosynthesis. The same tendency was observed for IF002 harboring *ldhA*_*Cd*_*-hadA*_*Cd*_ (data not shown).

### Identification of dehydrogenases responsible for 2HB oxidation in *R. eutropha*

In the genome of *R. eutropha* H16, there is no gene highly homologous to previously known 2-hydroxyacid dehydrogenases. We thus considered the possibility that the conversion of (*R*/*S*)-2HB to 2KB would be mediated by some homolog(s) of lactate dehydrogenases (LDHs). The probable LDHs identified in *R. eutropha* H16 are listed in Table 2 and supplementary Table S3, and their gene loci on the chromosomes are shown in supplementary Fig. S2. Individual deletion strains for these probable LDHs were constructed. It should be noted that the two adjacent genes encoding predicted d-LDHs, *h16_A1681* (*ldhA1*) and *h16_A1682* (*ldhA2*), have a completely identical nucleotide sequence to each other, due to duplication of 1676 bp regions of 1,834,348-1,836,023 and 1,836,024-1,837,699 on chromosome 1. The actual presence of the duplicated regions (or three repeats in some clones) in IF001 was confirmed by PCR analysis. The knockout of LdhA1 and LdhA2 was conducted by removing both *h16_A1681* and *h16_A1682* along with the intergenic region. The genes *h16_B0093*, *h16_B0092*, and *h16_B0091*, sharing 44.2%, 26.0%, and 40.1% identities to *lutA*, *lutC*, and *lutB* derived from *Bacillus subtilis*, respectively, are a homolog set of [Fe-S] cluster protein-dependent l-lactate utilization (LUT) system. It has been reported that the LUT system plays a major role in growth of *B. subtilis* on l-lactate (Chai et al. 2009). The LUT system-deficient derivative of *R. eutropha* IF001 was constructed by deletion of the gene cluster of *h16_B0093*-*B0092*-*B0091*.

**Table 2:** Lactate dehydrogenase (LDH) homologs in *R. eutropha* H16 genome

Locus tag	KEGG name	KEGG annotation	Electron carrier	Length [amino acids]
d-LDHs				
H16_A1681	LdhA1	d-Lactate dehydrogenase	NAD(P)H	331
H16_A1682	LdhA2	d-Lactate dehydrogenase	NAD(P)H	331
H16_A3091	Dld	d-Lactate dehydrogenase	Cytochrome	476
l-LDHs				
H16_A0666	Ldh	l-Lactate dehydrogenase	NAD(P)H	349
H16_B0460	LldA	l-Lactate cytochrome reductase	Cytochrome	381
H16_B1817	LldD	l-Lactate cytochrome c reductase	Cytochrome	391
H16_B0091	LldF	Iron-sulfur cluster-binding protein (l-Lactate dehydrogenase complex)	Unknown	481
H16_B0092	LldG	Conserved hypothetical protein (l-Lactate dehydrogenase complex)	Unknown	233
H16_B0093	LldE	Fe-S oxidoreductase (l-Lactate dehydrogenase complex)	Unknown	260

**Fig. 4:**
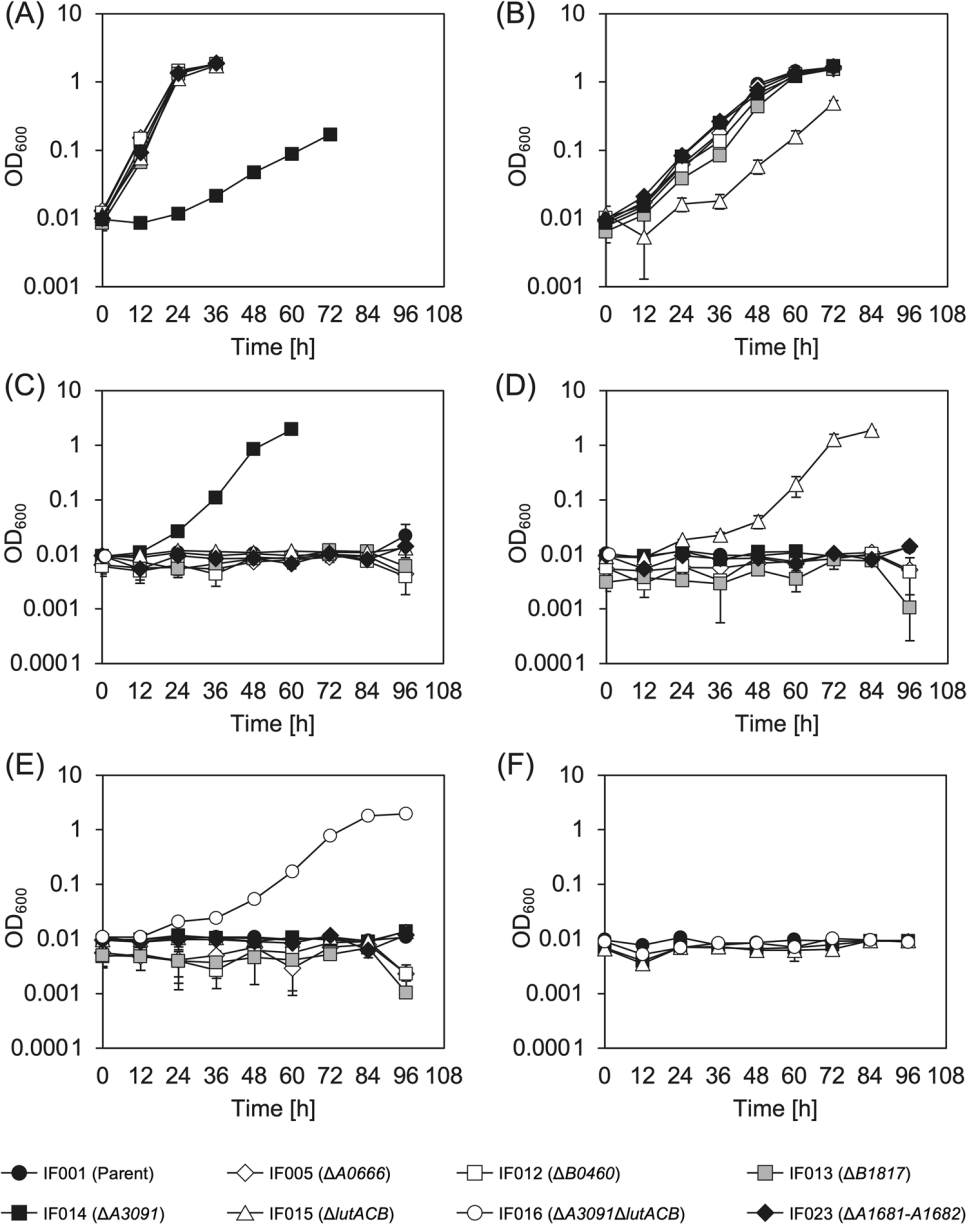
Growth of LDH-deleted strains of *R. eutropha* on 1% (w/v) sodium d-lactate (**A**), 1% (w/v) lithium l-lactate (**B**), and growth on 2% (w/v) glucose co-supplemented with 0.005% (w/v) sodium (*R*)-2HB (**C**), 0.005% (w/v) sodium (*S*)-2HB (**D**), 0.01% (w/v) sodium (*RS*)-2HB (**E**), or 0.01% (w/v) sodium 2KB (**F**). The *R. eutropha* strains were cultivated in a 3 mL phosphate-limited mineral salt medium containing the additives noted above at 30 °C with reciprocal shaking (170 strokes/min) (triplicate)

The resulting IF001-based strains IF005, IF012, IF013, IF014, IF015, IF016, and IF023 were cultivated in the phosphate-limited synthetic media containing d- or l-lactate as a sole-carbon source (Fig. 4A or B), as well as in the glucose media supplemented with (*R*)-, (*S*)-, or (*RS*)-2HB (Fig. 4C, D or E). Among the strains examined, only IF014 lacking H16_A3091 (Dld) showed severely poor growth on d-lactate (Fig. 4A), indicating the critical role of Dld in conversion of d-lactate to pyruvate. All the strains could grow on l-lactate, where IF015 lacking H16_B0093-B0092-B0091 (LutACB) exhibited lag-time in the growth (Fig. 4B). This suggested that LutACB partially participated in utilization of l-lactate although other enzyme(s) would support the growth on l-lactate. As expected, the lower growth ability on d- and l-lactate was related to resistance to (*R*)- and (*S*)-2HB, respectively. IF014 and IF015 could grow in the medium containing (*R*)-2HB and (*S*)-2HB, respectively, and the strain IF016 doubly lacking Dld and LutACB showed significant growth in the medium containing (*RS*)-2HB (Fig. 4E). These strains IF014, IF015, and IF016 were still sensitive to 2KB (Fig. 4F), which was consistent with the assumption that 2KB generated from (*R*/*S*)-2HB had the actual toxic property to the cells of *R. eutropha*.

### Biosynthesis of 2HB-containing copolymers by recombinant *R. eutropha*

The LDH-modified variants IF017 [∆*h16_A3091* (∆*dld*)], IF018 [∆*h16_B0093-B0092-B0091* (∆*lutACB*)], and IF019 [∆*h16_A3091*, *∆h16_B0093-B0092-B0091* (∆*dld*∆*lutACB*)] were re-constructed based on IF002 (*phaC*_*AR*_^+^, *ldhA*_*Cd*_*-hadA*_*Cd*_^*+*^) as the parent strain. These strains produced PHA up to 45–49 wt% on glucose, in which faint amount of 2HB unit (~0.1 mol%) was detected (entries 3–5 in Fig. 2 and supplementary Table S2). When 0.05% (w/v) l-valine was supplied along with glucose, IF017 and IF019 lacking Dld accumulated PHA containing 0.23 mol% 2HB fraction (entries 8 and 10 in Fig. 2 and supplementary Table S4). PHA production on glucose in the presence of 0.25% (w/v) (*RS*)-2HB precursor was performed with further co-supplementation of 0.05% (w/v) l-valine, because such the higher concentration of (*RS*)-2HB still caused growth defect even for the LDH-modified strains (data not shown). The PHA production was markedly reduced to 8–16 wt% by addition of (*RS*)-2HB (entries 11–15 in Fig. 2 and supplementary Table S5). The strain IF002 synthesized PHA composed of 3HB and 8.5 mol% of 2HB units (entry 12), demonstrating the actual ability of PhaC_*AR*_ to copolymerize 2HB-CoA with 3HB-CoA in *R. eutropha* when 2HB-CoA was sufficiently available. The 2HB fraction in PHA was increased up to 10.7 mol% by inactivation of Dld (entry 13), whereas the lack of LutACB did not alter the 2HB composition but unexpectedly increase 3HV fraction up to 6.2 mol% (entry 14). Double deletion of *dld* and *lutACB* resulted in higher composition of both 2HB (10.8 mol%) and 3HV (3.2 mol%) when compared to PHA synthesized by the parent strain IF002 (entry 15).

### Effects of overexpression of PhaC_*AR*_ on PHA biosynthesis

**Fig. 5:**
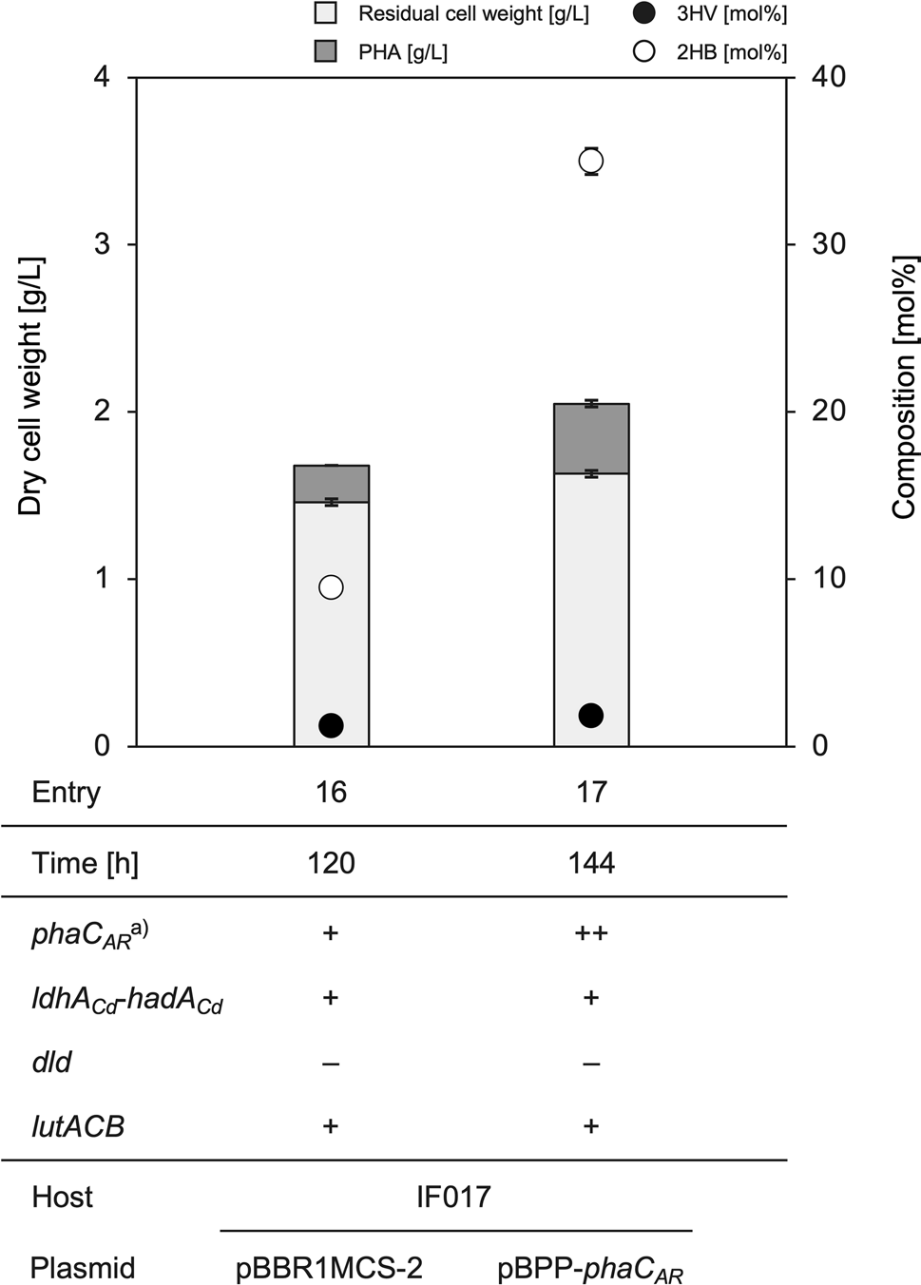
PHA biosynthesis by IF017 (*phaC*_*AR*_^+^, *ldhA*_*Cd*_-*hadA*_*Cd*_^+^, Δ*dld*) harboring pBBR1MCS-2 (entry 16), or pBPP-*phaC*_*AR*_ (entry 17) on 2% (w/v) glucose co-supplemented with 0.25% (w/v) sodium (*RS*)-2HB and 0.05% (w/v) l-valine. The residual cell weight and PHA production are shown in gray and dark gray bars, respectively, and 3HV and 2HB compositions are shown as black and white circles, respectively. The cells were cultivated in a 100 mL phosphate-limited mineral salt medium containing the additives described above with 100 μg/mL kanamycin for 120–144 h at 30 °C (triplicate). a) +, *phaC*_*AR*_ on chromosome; ++, *phaC*_*AR*_ on both chromosome and plasmid

The vector for overexpression of *phaC*_*AR*_ was constructed by inserting *phaC*_*AR*_ into a broad host range-expression vector pBPP at the downstream of *P*_*phaP*_. It has been demonstrated that *P*_*phaP*_ in pBPP acted as a strong constitutive promoter when introduced into *R. eutropha* (Fukui et al. 2011). In the phosphate-limited medium containing 2% (w/v) glucose co-supplemented with 0.25% (w/v) sodium (*RS*)-2HB and 0.05% (w/v) l-valine, the strain IF017/pBPP-*phaC*_*AR*_ accumulated PHA with 21 wt%, and the 2HB composition reached up to 35.0 mol% (Fig. 5 and supplementary Table S6). The molecular weights of the resulting 2HB-containing PHAs were comparable with those of PHAs synthesized by the engineered *E. coli* harboring *phaC*_*AR*_ (Table 3).

**Table 3:** Molecular weight of PHA synthesized by IF017-based strains of *R. eutropha*

Entry	Host	Plasmid	Composition [mol%]			Mw [× 10^5^]	PDI	Ref
			3HB	3HV	2HB			
16	*R. eutropha* IF017	pBBR1MCS-2	89.3 ± 0.2	1.2 ± 0.0	9.5 ± 0.2	6.6	1.4	This study
17		pBPP-*phaC*_*AR*_	63.2 ± 0.8	1.8 ± 0.2	35.0 ± 0.8	3.3	1.4	This study
	*E. coli* JM109	pBSP_*Re*_*phaC*_*AR*_*pct*	74.6^a)^	–	25.6 ± 5.0	3.7	1.9	Matsumoto et al. 2018
			59.4^a)^	–	40.6 ± 0.3	3.9	1.9	

*Mw*, weight-average molecular weight; *PDI*, poly dispersity index. a) Estimated from the 2HB composition

### Structural analyses of the 2HB-containing PHAs

**Fig. 6:**
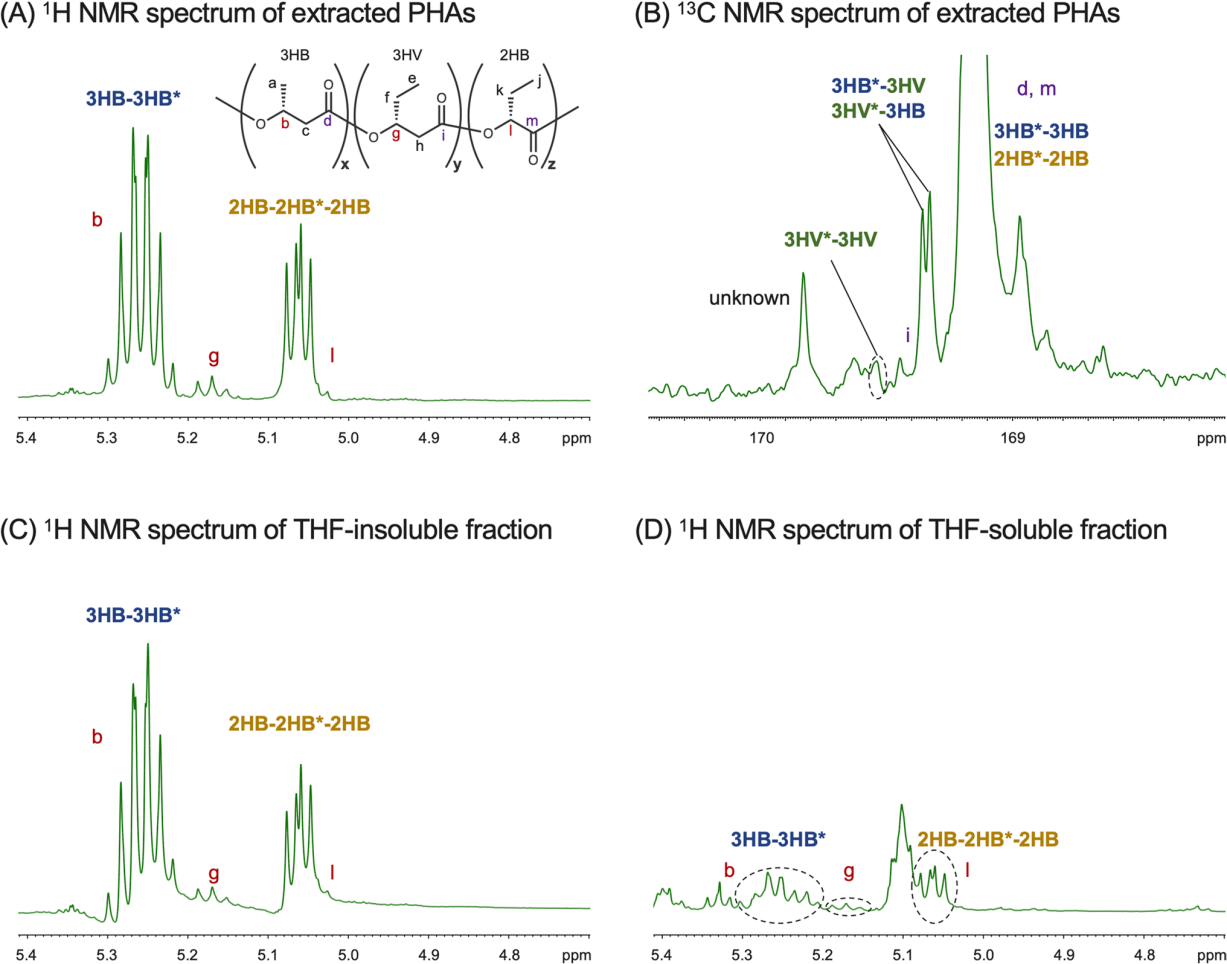
Verification of block-copolymerization property of PHAs synthesized by the engineered *R. eutropha* by solvent fractionation. ^1^H NMR (**A**) and ^13^C NMR (**B**) spectra of the PHAs extracted from IF017/pBPP-*phaC*_*AR*_, and ^1^H NMR spectra of the THF-insoluble fraction (**C**) and THF-soluble fraction (**D**). The methine proton regions in ^1^H NMR spectra and carbonyl carbon region in ^13^C NMR spectrum are shown

The PHA extracted from the dried cells of IF017/pBPP-*phaC*_*AR*_ (entry 17) was determined to be actually a terpolymer composed by 3HB, 3HV, and 2HB units by ^1^H NMR analysis (Fig. 6 and supplementary Fig. S3). It was reported that, when 2HB and 3HA (3HB and 3HV) units were randomly distributed in the polymer chain, the methine proton of 2HB unit showed a complex resonance pattern at 4.8–5.1 ppm based on four triad sequences of 3HA-2HB*-3HA, 3HA-2HB*-2HB, 2HB-2HB*-3HA, and 2HB-2HB*-2HB, and that of 3HB was also resolved at 5.2–5.4 ppm due to dyad sequences of 3HB-3HB* and 2HB-3HB* (Matsumoto et al. 2018). The methine proton resonances in the extracted PHA both showed single pattern of 2HB-2HB*-2HB and 3HB-3HB* (Fig. 6A and supplementary Fig. S3A), indicating that the hetero-linkages between 3HB and 2HB units were less than detectable level. The same resonance patterns for methine protons were also observed for PHA synthesized by IF017/pBBR1MCS-2 not overexpressing *phaC*_*AR*_ (supplementary Fig. S3B). The carbonyl carbon resonance in the ^13^C NMR spectrum of PHA synthesized by IF017/pBPP-*phaC*_*AR*_ was resolved into four peaks arisen from dyad sequences of 3HB and 3HV units. Although the signal corresponding to 3HV-3HV* homo-linkage was too small to calculate dyad-sequence distribution, the rather high signal intensities of the hetero-linkages of 3HB*-3HV and 3HV-3HB* strongly suggested high randomness of the distribution of 3HB and 3HV units (Fig. 6B and supplementary Fig. S4).

These results raised two possibilities that the PHAs synthesized by the recombinant *R. eutropha* were a block copolymer consisting of P(3HB-*co*-3HV) segment and P(2HB) segment [P(3HB-*co*-3HV)-*block*-P(2HB)], or blend of P(3HB-*co*-3HV) copolymer and P(2HB) homopolymer. We then examined solvent fractionation, because P(2HB) homopolymer in P(3HB)/P(2HB) blend was soluble in THF (Matsumoto et al. 2018). When about 40 mg of the polymer extracted from the cells of IF017/pBPP-*phaC*_*AR*_ was treated with THF, a large proportion was THF-insoluble while a very low amount of polymer (< 1 mg) was recovered in the THF-soluble fraction. The ^1^H NMR analysis of the THF-insoluble fraction detected the same resonances patterns of methine protons of 2HB, 3HB, and 3HV (Fig. 6C and supplementary Fig. S5A) as those of the polymer before the fractionation (Fig. 6A, supplementary Fig. S3A). It was notable that the methine protons of 3HB and 3HV units were still detected in the THF-soluble fraction along with probable methine resonance of 2HB-2HB*-2HB triad, although signals derived from unknown impurities were observed due to low recovery yield (Fig. 6D and supplementary Fig. S5B). Given that the THF-soluble fraction was not P(2HB) homopolymer, the PHA synthesized by the engineered *R. eutropha* is most likely a block copolymer consisting of P(3HB-*co*-3HV) and P(2HB) segments linked with a covalent bond.

## Discussion

This study focused on the application of the sequence-regulating chimeric PHA synthase PhaC_*AR*_ in the well-known PHA producer, *R. eutropha*. During the investigation, we observed a strong growth inhibitory effect of 2HB to *R. eutropha* cells (Fig. 3A). The biosynthesis of 2HB-containg PHAs by *R. eutropha* has been reported by using a mutant of PHA synthase derived from *Pseudomonas* sp. 6-19 (Park et al. 2013), in which PHA synthesis was done by growth-unassociated two-step cultivation in a nitrogen-free synthetic medium containing 2HB, and thus there was no description regarding the growth inhibition by 2HB. It was thought that the effect of 2HB on the growth was related to 2HB metabolism specific in *R. eutropha*, because the growth of recombinant *E. coli* harboring *phaC*_*AR*_ was not significantly impaired by addition of 0.5% (w/v) 2HB (Matsumoto et al. 2018).

The growth inhibition of *R. eutropha* by 2HB was attributed to l-valine deficiency, and the present results supported the assumption that 2KB was the actual inhibitor. l-Isoleucine and l-valine are biosynthesized *via* the shared pathway in which the first reaction is catalyzed by acetohydroxyacid synthase (AHAS) (supplementary Fig. S1). l-Isoleucine is the end product when 2KB and pyruvate are condensed by AHAS, while l-valine is synthesized *via* condensation of two molecules of pyruvate by AHAS. Generally, biosynthesis of branched-chain amino acids is tightly regulated by several factors. In *E. coli*, there are three AHAS isozyme, AHAS I, II, and III, encoded by *ilvBN*, *ilvGM*, and *ilvIH*, respectively. It was reported that AHAS II and III prefer to 2KB than pyruvate as the substrate, and *E. coli* K12-derived strains lack AHAS II due to flame shift mutations (Li et al. 2017). *R. eutropha* possesses a unique AHAS encoded by *ilvB* (*h16_A1035*) and *ilvH* (*h16_A1036*), which are clustered on chromosome 1 along with *ilvC* (*h16_A1037*) encoding acetohydroxyacid isomeroreductase (AHAIR). Lu et al. reported that IlvBH_*Re*_ showed 140-times higher catalytic selectivity towards 2KB than pyruvate, and was regulated through feedback inhibition by the branched-chain amino acids (Lu et al. 2015). It was plausible that the 2KB-preferring IlvBH_*Re*_ predominantly mediated the condensation of 2KB and pyruvate over the condensation of two molecules of pyruvate when intracellular concentration of 2KB was increased by oxidation of 2HB supplemented into the medium. This would lead to relatively lower flux of l-valine synthesis than that of l-isoleucine synthesis and consequent deficiency of l-valine. The feedback inhibition of IlvBH_*Re*_ by l-isoleucine, over-produced in the presence of 2HB, may further promote the l-valine depletion. The growth restoration by high concentration of pyruvate (Fig. 3C and D) might be because the intracellular concentration ratio of 2KB/pyruvate returned to the normal level.

The growth inhibition by the (*R*/*S*)-2HB-derived 2KB was based on the presence of endogenous dehydrogenation activity towards 2HB. We here found that Dld (H16_A3091) and LutACB (H16_B0093-B0092-B0091) played a role in assimilation of d- and l-lactate, and moreover, they also related to the cellular sensitivity against (*R*)- and (*S*)-2HB, respectively. These results demonstrated the metabolic functions of Dld and LutACB in dehydrogenation of (*R*)- and (*S*)-2-hydroxyacids of C_3_–C_4_, although the contribution of LutACB to the conversion of l-lactate to pyruvate was seemed to be partial (Fig. 4B). These are new knowledge regarding the specific properties of *R. eutropha* for metabolisms of short-chain-length 2-hydroxyacids.

Under the (*RS*)-2HB-supplemented condition, IF002 (*phaC*_*AR*_^+^, *ldhA*_*Cd*_-*hadA*_*Cd*_^*+*^) produced PHA composed of 8.5 mol% of 2HB fraction owing to the polymerization ability of PhaC_*AR*_ to 2HB-CoA (entry 12). Considering that PHA synthesized by IF001 (*phaC*_*AR*_^+^) contained no 2HB fraction even with the supplementation of (*RS*)-2HB (entry 11), *R. eutropha* does not have endogenous CoA transferase or CoA ligase activity to 2HB, thus the heterologous HadA_*Cd*_ functioned as the CoA transferase generating 2HB-CoA. This agreed with the previously reported characteristics of an endogenous propionate CoA transferase (H16_A2718) that showed no activity to 2HB despite the broad substrate specificity towards various acids including 3HB, lactate, and glycolate (Volodina et al. 2014). Meanwhile, the lack of Dld resulted in an increase in 2HB composition up to 10.7 mol% (entries 13 and 15), probably due to higher availability of (*R*)-2HB attributed to the weakened conversion of (*R*)-2HB to 2KB. The 2HB composition was further increased up to 35 mol% by introduction of pBPP-*phaC*_*AR*_. This was consistent with the previous observation that higher expression of PHA synthase tended to promote incorporation of the minor 3HHx unit in PHA (Dennis et al. 1998; Kawashima et al. 2015). The expression level of PHA synthase was an important factor for compositional regulation of the 2HB-containg PHA in *R. eutropha*.

There was no information regarding comonomer distribution and molecular weights of 2HB-containing PHAs synthesized by the previously reported *R. eutropha* strains expressing the mutant of pseudomonad PHA synthase. We here showed that PHAs synthesized by IF017/pBPP-*phaC*_*AR*_ in the presence of (*RS*)-2HB were most likely a block copolymer consisting of P(3HB-*co*-3HV) and P(2HB) segments, P[(3HB-*co*-3HV)-*block*-2HB]. The weight-average molecular weights were 3.3–6.6 × 10^5^, which were comparable with those synthesized by recombinant *E. coli* expressing PhaC_*AR*_. It was notable that PHA with altered 2HB composition synthesized by IF002 having intact *dld* also showed block sequence (data not shown). These results strongly suggested that the sequence-regulating polymerization was owing to the specific property of the chimeric PHA synthase, but independent from the metabolic background of the host cells.

At present, a very small fraction of 2HB unit was detected in PHA produced by the IF002-based *ldhA*_*Cd*_-*hadA*_*Cd*_^+^ strains when glucose was fed as a sole carbon source (entries 2–5). Considering the fact that strains not harboring *ldhA*_*Cd*_-*hadA*_*Cd*_ incorporated no 2HB unit into PHA on glucose in both and *E. coli* (Sudo et al. 2020) and *R. eutropha*, LdhA_*Cd*_ was functional to 2KB to some extent but the participation in the formation of (*R*)-2HB from 2KB would be insufficient, also as suggested above. Insufficient metabolic flux from l-threonine to 2KB would be another possible cause for the low 2HB fraction in PHA produced from glucose. Further engineering focusing on these points would be useful to establish *R. eutropha* strains capable of producing the 2HB-containing PHAs efficiently from structurally unrelated carbon sources.

### Supplementary information

ESM 1 (PDF 2.40 mb)

## Data Availability

The data generated during the study are included in this article and its supplementary material.
