# Supplementary material for: (R/S)-lactate/2-hydroxybutyrate dehydrogenases in and biosynthesis of block copolyesters by Ralstonia eutropha
Source: Appl Microbiol Biotechnol. 2023 Sep 29;107(24):7557–69. doi: 10.1007/s00253-023-12797-6 (PMC10656315; doi:10.1007/s00253-023-12797-6)
Supplement: Supplementary file 1 — (PDF 2.40 mb) [file 253_2023_12797_MOESM1_ESM.pdf]

Applied Microbiology and Biotechnology
Doi: 10.1007/s00253-023-12797-6

Additional file:

(*R*/*S*)-Lactate/2-hydroxybutyrate dehydrogenases in and biosynthesis of block copolyesters by *Ralstonia eutropha*

Shizuru Ishihara[1], Izumi Orita[1], Ken'ichiro Matsumoto[2], Toshiaki Fukui[1]

1) School of Life Science and Technology, Tokyo Institute of Technology, 4259 Nagatsuta, Midori-ku, Yokohama 226-8501, Japan
2) Division of Biotechnology and Macromolecular Chemistry, Graduate School of Engineering, Hokkaido University, N13W8, Kita-ku, Sapporo 060-8628, Japan

*Corresponding author
Toshiaki Fukui
School of Life Science and Technology
Tokyo Institute of Technology
B-37 4259 Nagatsuta, Midori-ku, Yokohama 226-8501, Japan
Tel/Fax: +81-45-924-5766
e-mail: tfukui@bio.titech.ac.jp

Supporting Information

Fig. S1
Fig. S2
Fig. S3
Fig. S4
Fig. S5
Table S1
Table S2
Table S3
Table S4
Table S5
Table S6

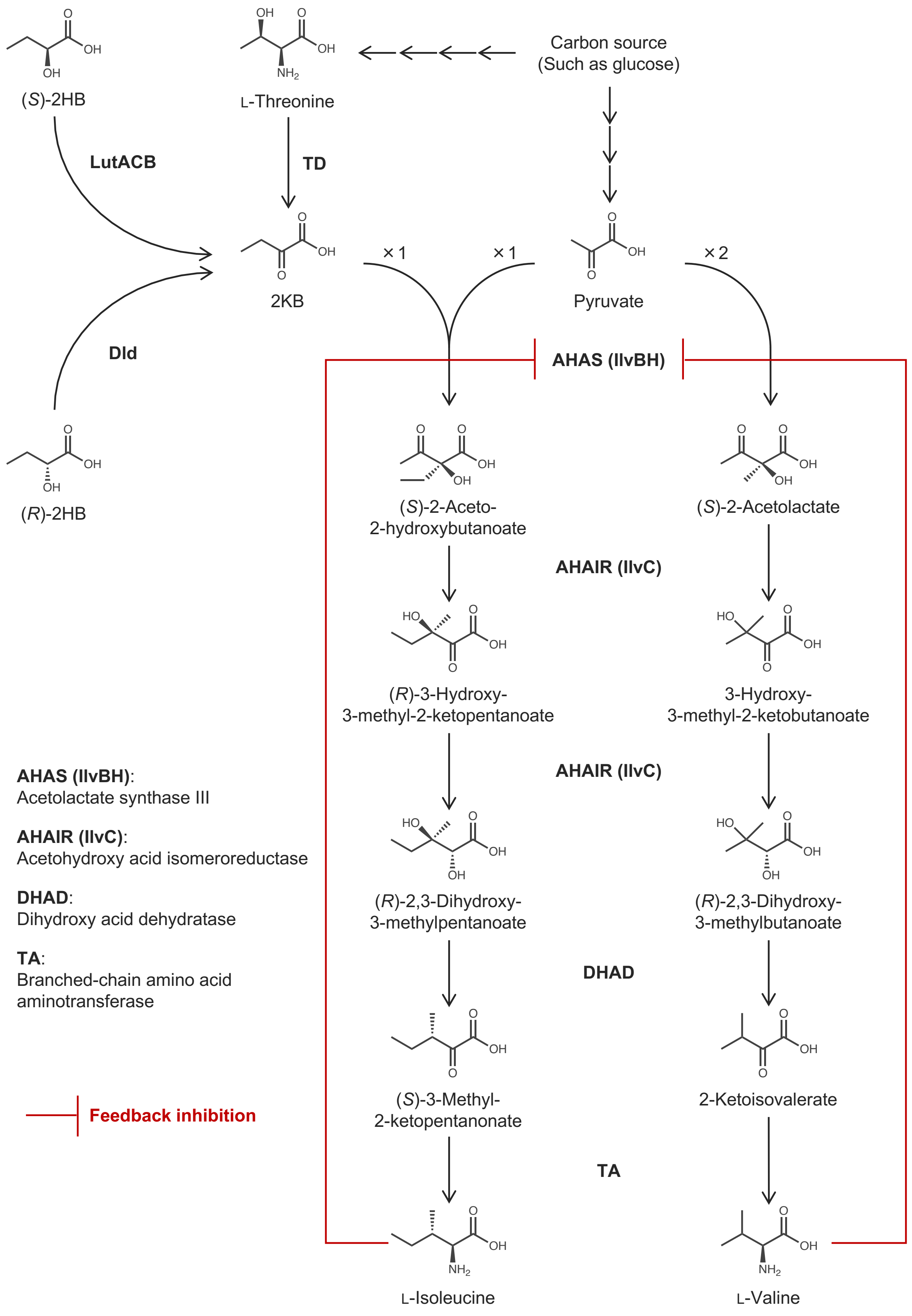

Fig. S1. The biosynthesis pathway of L-isoleucine and L-valine in *R. eutropha*.
TD, L-threonine dehydratase; Dld, cytochrome-dependent D-lactate dehydrogenase; LutACB, [Fe-S] cluster protein-dependent L-lactate utilization system; AHAS (IlvBH), acetolactate synthase III; AHAIR (IlvC), acetohydroxy acid isomeroreductase; DHAD, dihydroxy acid dehydratase; TA, branched-chain amino acid aminotransferase.

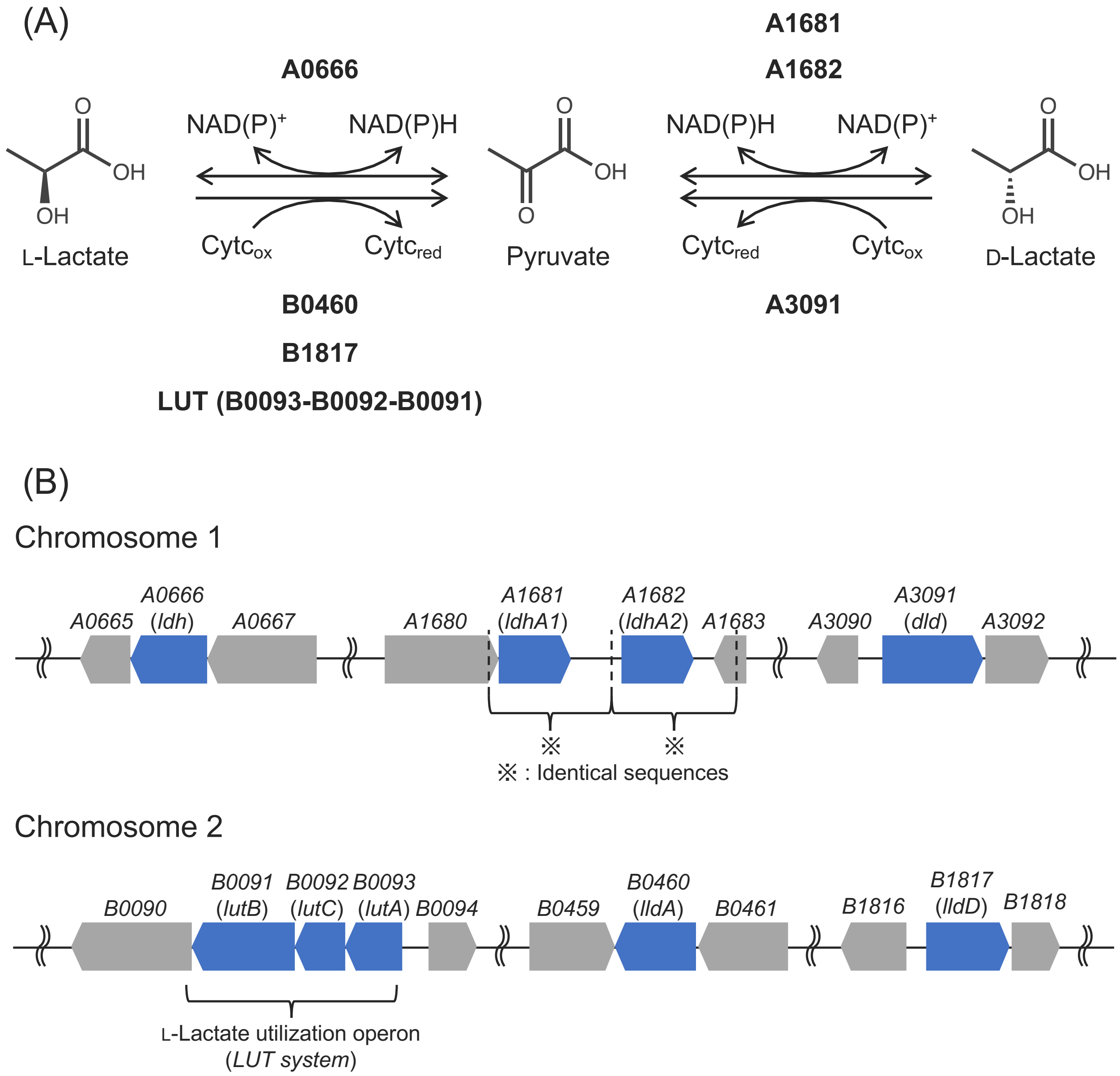

Fig. S2. Predicted reactions catalyzed by D/L-lactate dehydrogenase homologs in *R. eutropha* H16 (A), and their gene loci on the chromosomes (B). A0665, putative bifunctional transglycosylase and transpeptidase; A0666, NAD(P)H-dependent L-lactate dehydrogenase; A0667, sodium: sulfate symporter transmembrane region; A1680, predicted permease; A1681 and A1682, NAD(P)H-dependent D-lactate dehydrogenases; A1683, predicted acetyltransferase; A3090, cobalamin adenosyltransferase; A3091, cytochrome-dependent D-lactate dehydrogenase; A3092, transcriptional regulator, LysR family; B0090, lactate permease; B0091, L-lactate dehydrogenase complex, iron-sulfur cluster-binding protein (LldF/LutB)); B0092, L-lactate dehydrogenase complex, conserved hypothetical protein (LldG/LutC); B0093, L-lactate dehydrogenase complex, Fe-S oxidoreductae protein (LldE/LutA); B0094, transcriptional regulator, GntR-family; B0459, acetylornithine deacetylase; B0460, cytochrome-dependent L-lactate dehydrogenase; B0461, predicted flavoprotein involved in K^+ transport; B1816, transcriptional regulator, LysR family; B1817, cytochrome-dependent L-lactate dehydrogenase; B1818, demethylmenaquinone methyltransferase.

(A) PHA synthesized by IF017/pBPP-*phaC*$_{AR}$

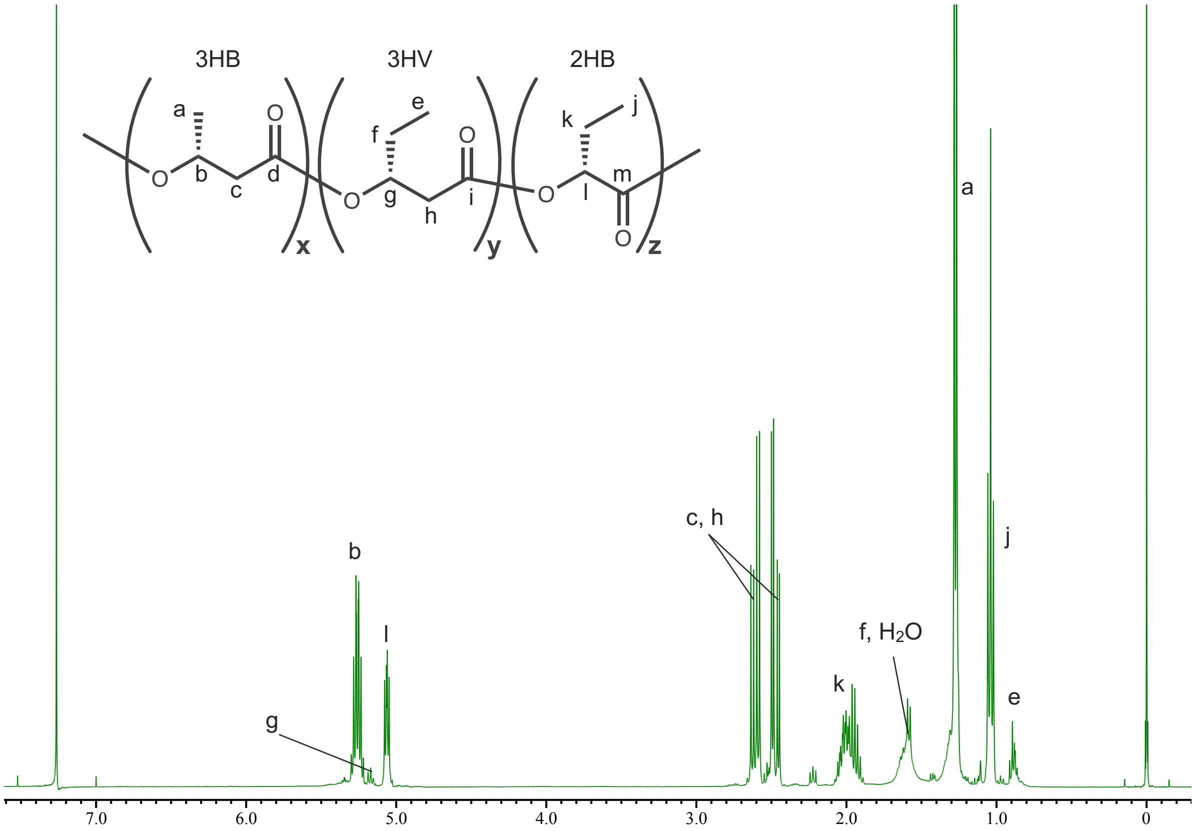

(B) PHA synthesized by IF017/pBBR1MCS-2

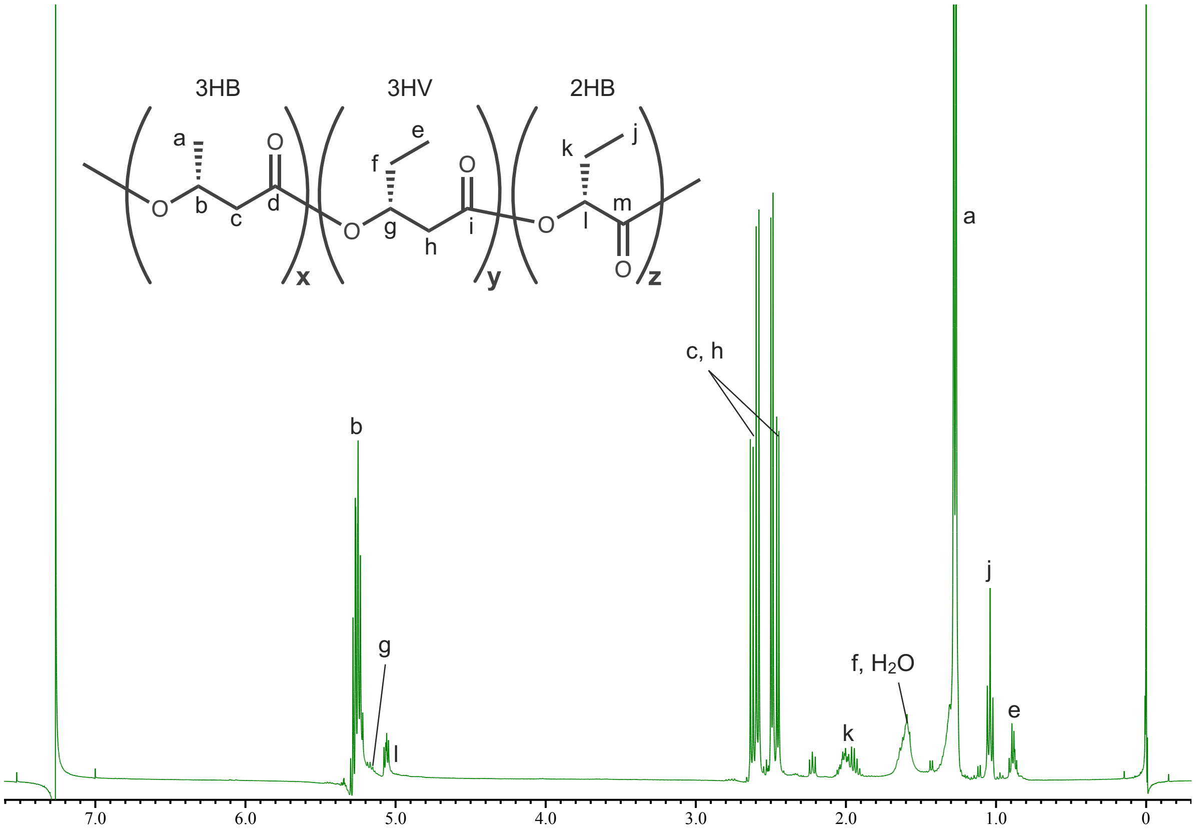

Fig. S3. ^{1}H NMR analysis of the PHAs synthesized by *R. eutropha* IF017/pBPP-*phaC*$_{AR}$ (A) and IF017/pBBR1MCS-2 (B).

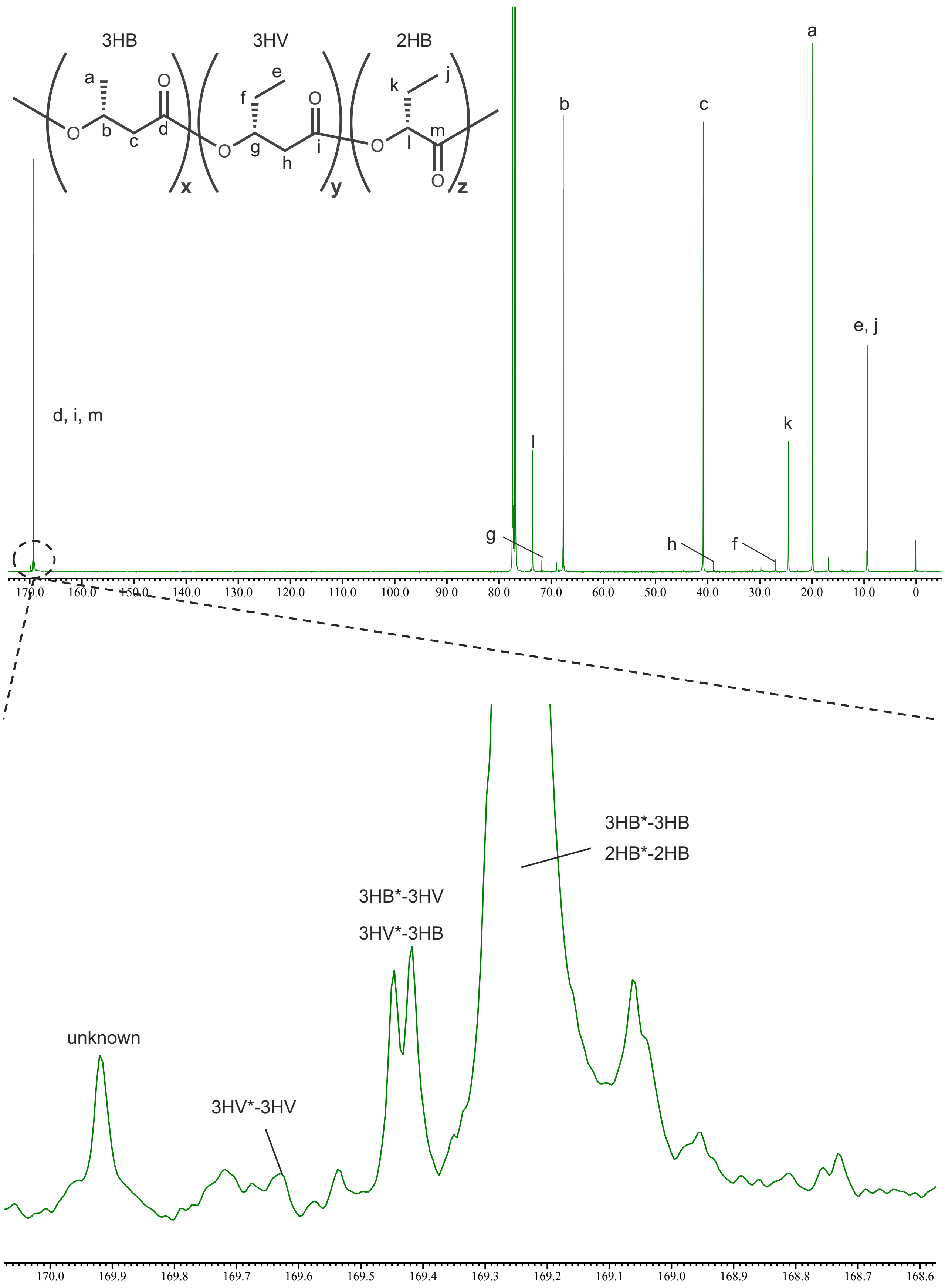

Fig. S4. ^{13}C NMR analysis of the PHA synthesized by *R. eutropha* IF017/pBPP-*phaC*$_{AR}$.

(A) THF-insoluble fraction

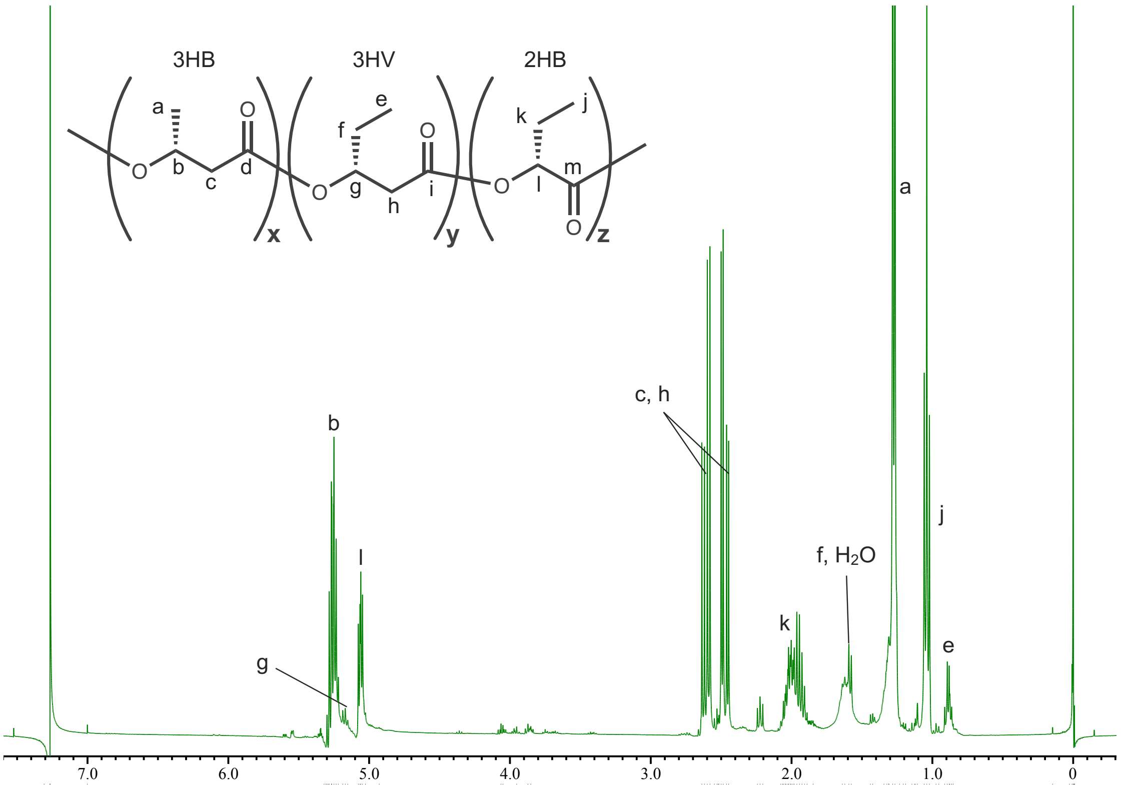

(B) THF-soluble fraction

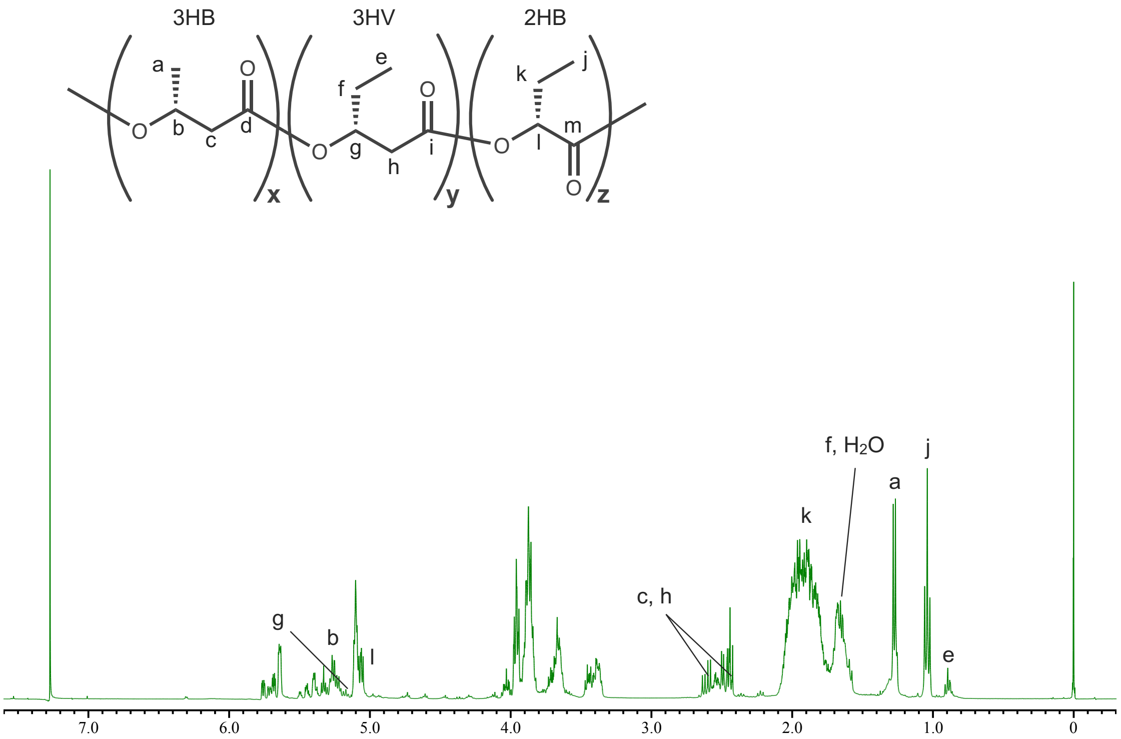

Fig. S5. Verification of block-copolymerization property of PHAs synthesized by the engineered *R. eutropha* by solvent fractionation. Full spectra of ^{1}H NMR of the THF-insoluble fraction (A) and soluble fraction (B) of PHA extracted from IF017/pBPP-$phaC_{AR}$.

Table S1. Primers used in this study.

Name	Sequence (5' → 3')	Note
phaCar_Fw	AATAGTGACGGCAGAGAGACAATCAAATCATGAGCC AACCATCTTATGGCCCGC	
phaCar_Rv	TGCAGGCCTGCCGGCGCCGTGCATATGCAAGCGTC ATGCCTTGGCTTTGACGTATCGC	For construction of pK18ms-C*ar*AB'
pK18msCAB-invR	GCCAGGGCAATGCCCGGAGCCGGTTCG	
pK18msCAB-invF	CCCTCCCGTTTCCATTGAAAGGA	
pEE32R-CAcup-inv	GTGCTCTCCTTCACCCACACCCGA	
phaPRe-inv2	TAACTGCCTGCGTTGAAGATGGAC	
ldhA-hadA-inf_Fw	CTCGGGTGTGGGTGAAGGAGAGCACATGAAAATCC TGGTGTTTGGAGCG	For construction of pK18ms-P1udP1-ldhA*Cd*hadA*Cd*
ldhA-haA-inf_Rv	GGTCCATCTTCAACGCAGGCAGTTATTAATAGCGGA CCACTTTACTGTC	
H16_A0666_ud1000_XbaI_Fw	GCTCTAGACGCAGTGCCTGCGTGGTGCCGA	
H16_A0666_ud1000_XbaI_Rv	GCTCTAGACAGCGTTCGCTCAGGCGGCT	For construction of pK18ms-A0666ud1000
A0666ud1000-inv1	GGTTCGTACCTCCTCAGAC	
A0666ud1000-inv2	AGGTTCCGCCTGCGGCGGGACTCG	
H16_B0460_ud980_XbaI_Fw	GCTCTAGATGGTTGCCGCAACCGGCCCGTTCC	
H16_B0460_ud980_HindIII_Rv	CCCAAGCTTGGTGGAAGGCCAGCCCTGGACTTCAC	For construction of pK18ms-B0460ud980
B0460ud980-inv1	GGGAACTCCCTTGAAAAAAACAAAAAGCTGCCG	
B0460ud980-inv2	TAGCGTCTATGAGGCGTCCGGGCCGAAGCTGGCC	
H16_B1817_ud980_XbaI_Fw	GCTCTAGACCTTGGCATGAAGGCGAAACC	
H16_B1817_ud980_HindIII_Rv	CCCAAGCTTATCGATGTCGATGGTGTCGCAGC	For construction of pK18ms-B1817ud980
B1817ud980-inv1	TTGGCGGCGCAAGGTACCTGCGCCGCTTGC	
B1817ud980-inv3	GACATCGGCCCTGCCTGCATCC	
H16_A3091_ud980_XbaI_Fw	GCTCTAGAGGCAAATCCGAGTAATTTTCTCG	
H16_A3091_ud980_HindIII_Rv	CCCAAGCTTGCCGTGCAGCCGCAGCGCCTGGAAGG	For construction of pK18ms-A3091ud980
A3091ud980-inv1	GCGGGTCTCCGGCGGGTCGGCGCGACGGTG	
A3091ud980-inv2	GGCGTGCCGAGCTGATGGCCTCCATGTTCAACC	
H16_B0091-B0092-B0093_ud980_XbaI_Fw	GCTCTAGATGTTCGGCATCCCGGGCGTGAT	
H16_B0091-B0092-B0093_ud980_HindIII_Rv	CCCAAGCTTAGCCGCCGCCGAAGCCAGCCAGCG	For construction of pK18ms-B0091-B0092-B0093ud980
B0091-B0092-B0093ud980-inv1	GTCTCGCTCCGGACGTGCTGGGGTCGCACG	
B0091-B0092-B0093ud980-inv2	TCCGTCATCACGGGCGCGAGCGGTT	
H16_A1681-A1682_up-inf_Fw	TCGAGCTCGGTACCCTGCTGCGTGAAGCCACCTCG CTGTGC	
H16_A1681-A1682_up-inf_Rv	GGCCAACAGCCGTGTGATGTGTCTCCGCGGCGAAT CGC	For construction of pK18ms-A1681-A1682ud900
H16_A1681-A1682_down-inf_Fw	CCGCGGAGACACATCACACGGCTGTTGGCCTCAAA TCG	
H16_A1681-A1682_down-inf_Rv	CTCTAGAGGATCCCCGCCACCGGCTGACGCAGGCA AGGGAC	
pBPP-phaCar-inf_Fw	GGAGGTATATACATATGAGCCAACCATCTTATGGCC	For construction of pBPP-*phaCAR*
pBPP-phaCar-inf_Rv	CGACTCTAGAGGATCTCATGCCTTGGCTTTGACGTA TCGC	

Table S2. PHA production by engineered *R. eutropha* strains on glucose as sole carbon source.

Entry	Strain	Dry cell weight [g/L]	Residual cell weight [g/L]	PHA [g/L]	PHA content [wt%]	Composition [mol%]		
						3HB	3HV	2HB
1	IF001	2.86 ± 0.14	1.73 ± 0.05	1.13 ± 0.19	39.35 ± 4.71	99.89 ± 0.02	0.11 ± 0.02	Not detected
2	IF002	2.90 ± 0.05	1.59 ± 0.05	1.31 ± 0.04	45.24 ± 1.21	99.86 ± 0.01	0.07 ± 0.00	0.07 ± 0.00
3	IF017	2.71 ± 0.06	1.37 ± 0.06	1.34 ± 0.04	49.46 ± 1.39	99.88 ± 0.00	0.05 ± 0.01	0.07 ± 0.00
4	IF018	2.92 ± 0.01	1.47 ± 0.10	1.45 ± 0.11	49.55 ± 3.66	99.92 ± 0.00	0.05 ± 0.00	0.03 ± 0.00
5	IF019	2.65 ± 0.03	1.36 ± 0.03	1.29 ± 0.04	48.58 ± 1.10	99.86 ± 0.01	0.05 ± 0.01	0.09 ± 0.01

The cells were cultivated in a 100 mL phosphate-limited mineral salt medium containing 2% (w/v) glucose for 72 h at 30˚C.
All cultivations were performed in triplicate, and the data are expressed as the mean ± standard deviation.

Table S3. Similarities of lactate dehydrogenase (LDH) homologs in *R. eutropha*.

Queries	Hits	KEGG annotation	Identity / overlap
**D-LDHs**			
H16_A1681	b1380[a)]	D-Lactate dehydrogenase (LdhA)	50.3% / 328 a.a.
H16_A1682	b1380[a)]	D-Lactate dehydrogenase (LdhA)	50.3% / 328 a.a.
H16_A3091	197257[b)]	Lactate dehydrogenase D (LDHD)	49.7% / 477 a.a.
**L-LDHs**			
H16_A0666	b0801[a)]	Hydroxycarboxylate dehydrogenase B (HcxB)	37.5% / 323 a.a.
H16_B0460	b3605[a)]	L-Lactate dehydrogenase (LldD)	44.1% / 381 a.a.
H16_B1817	b3605[a)]	L-Lactate dehydrogenase (LldD)	37.2% / 371 a.a.
H16_B0091	BSU34040[c)]	Lactate utilization protein B (LutB, YvfW)	40.4% / 474 a.a.
H16_B0092	BSU34030[c)]	Lactate utilization protein C (LutC, YvbY)	26.0% / 123 a.a.
H16_B0093	BSU34050[c)]	Lactate utilization protein A (LutA, YvfV)	44.2% / 240 a.a.

a) from *Escherichia coli* K12 MG1655, b) from *Homo sapiens* (human), c) from *Bacillus subtilis* subsp. *subtilis* 168

Table S4. PHA production by engineered *R. eutropha* strains on glucose supplemented with L-valine.

Entry	Strain	Dry cell weight [g/L]	Residual cell weight [g/L]	PHA [g/L]	PHA content [wt%]	Composition [mol%]		
						3HB	3HV	2HB
6	IF001	3.83 ± 0.05	2.19 ± 0.31	1.64 ± 0.34	42.73 ± 8.53	99.87 ± 0.01	0.13 ± 0.01	Not detected
7	IF002	3.30 ± 0.18	1.59 ± 0.05	1.71 ± 0.22	51.65 ± 3.82	99.78 ± 0.01	0.12 ± 0.01	0.11 ± 0.01
8	IF017	2.88 ± 0.07	1.57 ± 0.05	1.31 ± 0.02	45.62 ± 0.54	99.64 ± 0.02	0.13 ± 0.01	0.23 ± 0.02
9	IF018	3.30 ± 0.07	1.66 ± 0.04	1.64 ± 0.07	49.63 ± 1.39	99.79 ± 0.01	0.10 ± 0.01	0.11 ± 0.01
10	IF019	2.70 ± 0.07	1.57 ± 0.06	1.13 ± 0.04	41.96 ± 1.26	99.65 ± 0.03	0.12 ± 0.01	0.23 ± 0.03

The cells were cultivated in a 100 mL phosphate-limited mineral salt medium containing 2% (w/v) glucose supplemented with 0.05% (w/v) L-valine for 96 h at 30˚C.

All cultivations were performed in triplicate, and the data are expressed as the mean ± standard deviation.

Table S5. PHA production by engineered *R. eutropha* strains on glucose supplemented with sodium (*RS*)-2HB and L-valine.

Entry	Strain	Dry cell weight [g/L]	Residual cell weight [g/L]	PHA [g/L]	PHA content [wt%]	Composition [mol%]		
						3HB	3HV	2HB
11	IF001	1.91 ± 0.04	1.59 ± 0.03	0.32 ± 0.01	16.64 ± 0.37	97.60 ± 0.10	2.40 ± 0.10	Not detected
12	IF002	1.54 ± 0.05	1.30 ± 0.04	0.24 ± 0.02	15.84 ± 0.55	89.23 ± 0.54	2.30 ± 0.02	8.47 ± 0.53
13	IF017	1.57 ± 0.03	1.36 ± 0.04	0.21 ± 0.01	13.36 ± 0.71	88.19 ± 0.26	1.12 ± 0.13	10.68 ± 0.17
14	IF018	1.18 ± 0.02	1.03 ± 0.01	0.15 ± 0.01	12.63 ± 1.00	85.14 ± 0.16	6.01 ± 0.27	8.85 ± 0.23
15	IF019	1.23 ± 0.01	1.11 ± 0.00	0.13 ± 0.00	10.45 ± 0.16	86.10 ± 0.29	3.17 ± 0.20	10.73 ± 0.44

The cells were cultivated in a 100 mL phosphate-limited mineral salt medium containing 2% (w/v) glucose supplemented with 0.25% (w/v) sodium (*RS*)-2HB and 0.05% (w/v) L-valine for 120 h at 30˚C.

All cultivations were performed in triplicate, and the data are expressed as the mean ± standard deviation.

Table S6. PHA production by $PhaC_{AR}$-overexpressed *R. eutropha* strain on glucose supplemented with sodium (*RS*)-2HB and L-valine.

Entry	Host	Plasmid	Time [h]	Dry cell weight [g/L]	Residual cell weight [g/L]	PHA [g/L]	PHA content [wt%]	Composition [mol%]		
								3HB	3HV	2HB
16	IF017	pBBR1MCS-2	120	1.68 ± 0.02	1.46 ± 0.02	0.22 ± 0.00	13.11 ± 0.30	89.29 ± 0.18	1.23 ± 0.05	9.48 ± 0.17
17		pBPP-*phaC*$_{AR}$	144	2.05 ± 0.03	1.63 ± 0.02	0.42 ± 0.02	20.64 ± 0.71	63.18 ± 0.82	1.83 ± 0.16	34.99 ± 0.78

The cells were cultivated in a 100 mL phosphate-limited mineral salt medium containing 2% (w/v) glucose with 0.25% (w/v) sodium (*RS*)-2HB and 0.05% (w/v) L-valine at 30˚C.

All cultivations were performed in triplicate, and the data are expressed as the mean ± standard deviation.
